# Hidden cities, hidden gaps: measuring facility readiness for maternal and newborn health services and its association with person-centred maternity care in sub-Saharan Africa’s urban informal settlements

**DOI:** 10.7189/jogh.16.04343

**Published:** 2026-09-25

**Authors:** Safia S Jiwani, Martin K Mutua, Kadari Cisse, Choolwe Jacobs, Anne Njeri, Mwiche Musukuma, Godfrey Adero, Ashley Sheffel, Melinda K Munos, Elizabeth Stierman, Cheikh Faye, Ties Boerma, Agbessi Amouzou

**Affiliations:** 1 Department of International Health, Johns Hopkins Bloomberg School of Public Health, Baltimore, Maryland, USA; 2 African Population and Health Research Center, East Africa Office, Nairobi, Kenya; 3 Institut de Recherche en Sciences de la Sante, Centre National de Recherche Scientifique et Technologique, Ouagadougou, Burkina Faso; 4 Department of Epidemiology and Biostatistics, School of Public Health, University of Zambia, Lusaka Zambia; 5 Global Financing Facility for Women Children and Adolescents, The World Bank, Washington DC, USA; 6 African Population and Health Research Center, West Africa Office, Dakar, Senegal; 7 Institute of Global Public Health, University of Manitoba, Winnipeg, Manitoba, Canada

**Keywords:** facility readiness, maternal and newborn health, person-centred maternity care, sub-Saharan Africa, urban health, informal settlements

## Abstract

**Background:**

In sub-Saharan Africa, maternal and newborn deaths remain disproportionately higher among low-income populations, and they are associated with delivery in poorly equipped facilities and a shortage of staff to manage birth complications. We measured facility readiness to provide essential maternal and newborn health services and its association with women’s experience of person-centred maternity care (PCMC), and we compared facilities serving and not serving informal settlements in Nairobi, Lusaka and Ouagadougou cities.

**Methods:**

We conducted a health facility assessment in public and private facilities serving select urban informal settlements in Nairobi, and we used existing data in Lusaka and Ouagadougou. We computed readiness indices for labour and delivery care, and small and/or sick newborn care (SSNC) in each city, and used *t* tests to compare them across facilities serving and not serving informal settlements. We linked women’s self-reported PCMC scores to the labour and delivery readiness score of the facility they attended and ran 2-level linear regression models testing the association between facility readiness and PCMC scores.

**Results:**

Facility readiness scores were computed among 18, 38, and 138 facilities offering delivery services in Nairobi, Lusaka and Ouagadougou respectively. Mean labour and delivery readiness scores in facilities serving informal settlements ranged from 55.9% in Ouagadougou to 73.6% in Lusaka; SSNC readiness ranged from 37.2% in Ouagadougou to 61.3% in Nairobi. While facilities serving informal settlements had statistically significantly poorer readiness in Lusaka and Ouagadougou, key items such as newborn caps, registers, guidelines, and staff trained in Kangaroo Mother Care were lacking across both areas. We found no significant association between facility readiness and PCMC.

**Conclusions:**

All facilities have substandard readiness for essential maternal and newborn health services, but those serving informal settlements are more disadvantaged. Investments in service readiness and quality of care remain critical.

The rapid growth of cities has been identified as one of the most pressing global health issues of the 21st century [1]. In sub-Saharan Africa (SSA), six in 10 people are projected to live in cities by 2050 [2]. Large rural-to-urban migration has led to the expansion of urban informal settlements, threatening governments’ capacity to ensure universal access to essential health services. While urban living exposes individuals to better employment, education, health and living conditions, the urban advantage is not equitably distributed among urban residents. This has created ‘hidden cities’ [1] of urban poor populations that no longer benefit from the urban advantage [3,4].

The challenges posed by rapid urbanisation are reflected in maternal and newborn health outcomes [5,6], a staggering 70% of global maternal deaths and almost a third of livebirths occur in sub-Saharan Africa [7,8], and evidence suggests that most deaths in LMICs are due to poor quality of care, rather than inadequate access to services [9]. Most maternal and newborn deaths occur during the intrapartum and immediate postpartum period, and are associated with unskilled birth attendance, delivery in poorly equipped facilities and a shortage of essential obstetric care supplies to manage birth complications [10]. Birth asphyxia, preterm births and infections account for 87% of newborn deaths in SSA [11], and can be prevented by ensuring access to high quality life-saving interventions [12], such as thermal protection of preterm babies and specialised treatment for newborns born small (premature or with low birthweight) or sick [13]. However, these services are not equitably available to all populations in need, and their quality remains variable.

Measuring quality of care, a complex multidimensional concept that goes beyond mere access to services, is therefore essential to identify gaps and target interventions effectively. The World Health Organization’s quality of care framework for maternal and newborn health [14], stemming from the Donabedian model [15], encompasses three core dimensions: provision of care, experience of care, and structural quality, also known as facility readiness. These dimensions not only shape health outcomes but also influence the long-term trust and engagement of urban populations with the healthcare system.

Facility readiness is defined as the capacity of a health facility to provide services following standard guidelines, based on the observed availability and functionality of key items such as equipment, commodities, amenities, standard precautions, the presence of trained staff, as well as appropriate systems in place to support high quality of care and safety [14]. It reflects cross-cutting physical and human resources and is a crucial pre-requisite for enabling high quality services. Facility readiness is generally measured through a facility inventory in health facility assessments (HFA), such as the Demographic and Health Surveys (DHS) programme’s Service Provision Assessment (SPA) [16] focusing on antenatal, family planning, maternity care and sick child services, as well as the WHO’s Service Availability Readiness Assessment (SARA) [17] and Harmonized Health Facility Assessment (HHFA) [18] covering a wider range of health services.

Patient experience of care is another core quality of care dimension and is typically measured through client exit surveys conducted upon discharge from facility care. Person-centred maternity care (PCMC) is one such measure referring to maternity care that is respectful of and responsive to women and their families’ preferences, needs and values, and ensuring that their values guide clinical decisions [19]. Afulani *et al.* have developed and validated a 30-item PCMC scale covering elements of patient dignity and respect, communication and autonomy, and supportive care [20,21].

While quality of care is generally explored nationally or sub-nationally, little is known about the quality of services among the most vulnerable urban populations, and differences within urban areas. Our study objectives were to:

• measure facility readiness for labour and delivery care and small and/or sick newborn care (SSNC) services in Nairobi, Lusaka and Ouagadougou’s urban informal settlements,

• compare service readiness between facilities serving and not serving urban informal settlements in Lusaka and Ouagadougou, and

• assess the associations between labour and delivery care readiness and PCMC in informal settlements in each city.

We hypothesised that women attending a facility with higher service readiness report a higher PCMC experience.

## METHODS

### Study setting

This study was conducted within select informal settlements in Nairobi, Lusaka and Ouagadougou cities. These were locally defined geographic areas housing the urban poorest women in each city. Informal settlements dwellers typically lack tenure security, basic services and infrastructure, and their housing does not comply with safety regulations, leading to hazardous living conditions [22]. In Nairobi, we selected the Korogocho and Viwandani urban informal settlements based on existing research activities [23]; in Lusaka and Ouagadougou, the existing health facility data covered all facilities including those serving and not serving the cities’ informal settlements. Importantly, each context was unique and the characteristics of informal settlements and their residents differed across sites. Details on the study setting in each city are reported elsewhere.[24]

### Study design and data

We conducted a study evaluating the quality of maternal and newborn health services in select urban informal settlements in Nairobi, Lusaka and Ouagadougou cities. We administered in-depth interviews with health providers, client exit surveys of women discharged from childbirth care, and health facility assessments for maternal and newborn health services between January and March 2023. This analysis focused on the health facility assessments, though we also used data from client exit surveys.

In Nairobi, we conducted a cross-sectional survey of health facilities offering maternal and newborn health services and primarily serving the Korogocho and Viwandani informal settlements. In Ouagadougou and Lusaka, we used existing data from the WHO’s Harmonized Health Facility Assessment (HHFA) [18] conducted in 2020/2021 and 2021/2022 respectively. Aside from minor country-specific adaptations, the variables were identical across countries. The HHFA are comprehensive health facility surveys that measure service availability and the capacity of facilities to meet standards of quality; they typically cover modules relating to service availability, service readiness, quality of care and management/finance. In Lusaka and Ouagadougou, these HHFA were conducted in a census of all health facilities, including those serving and not serving urban informal settlements [25,26]. In Lusaka, we defined facilities serving informal settlements as those located within an informal settlement; in Ouagadougou, given the small number of facilities located within informal settlements, they were defined as those located within a 1 km radius of *non-loti* (informal) areas.

As part of this study, we also administered client exit surveys to a random sample of 1,249 women of reproductive age (15–49 years) discharged from childbirth care in health facilities serving a subset of urban informal settlements in Nairobi, Lusaka and Ouagadougou. The survey included a validated 30-item PCMC scale covering dignity and respect, communication and autonomy, and supportive care subscales [20]. Additional details on the design and sampling of client exit surveys are reported elsewhere [24].

### Tool development and training

In Nairobi, we adapted the HHFA tool (version 1, September 2022) to the Kenyan context and restricted it to the following modules relevant for maternal and newborn health services: staffing and resources, infection prevention, outpatient maternal and newborn health services, delivery and newborn care services, laboratory, consumable commodities and pharmaceutical commodities. The tool was administered on a tablet and programmed using Survey CTO. Four clinicians were trained on the data collection process, and the tool was pre-tested during a pilot study. In Lusaka and Ouagadougou, the complete HHFA tool covering all service areas was implemented by WHO and in-country partner institutions [25,26].

### Sample size

All functional health facilities of allopathic medicine (public, private for-profit and private non-profit/religious) offering maternal and newborn health services were eligible for the study. In Nairobi, we restricted the sample to facilities serving the Korogocho and Viwandani informal settlements, defined based on country classifications. A mapping exercise and formative research identified 43 eligible health facilities located within or immediately neighboring the informal settlements, of which 38 agreed to participate. From the HHFA data, we identified 115 facilities serving and 215 facilities not serving informal settlements in Ouagadougou, and 31 facilities serving and 88 facilities not serving informal settlements in Lusaka. We excluded tertiary-level referral and specialised hospitals, as there were very few and they served the whole city, to allow for a better comparison between facilities serving and not serving informal settlements in Lusaka and Ouagadougou.

### Measures

#### Maternal and newborn health service readiness

This analysis focused on facility readiness for normal deliveries, as well as routine and special newborn care for small and/or sick newborns, reflecting the basic capacity needed in primary care facilities before referral to higher-level ones. Service readiness was defined based on the availability and functionality of key items needed to offer high quality care. We generated binary variables for each item, assigning a value of 1 if the item was observed available and functional on the day of the survey, and 0 otherwise. For labour and delivery care readiness, we generated a simple additive readiness score based on the number of items available and functional to provide high-quality service; each item therefore carried equal weight towards the overall score. Item selection was based on HHFA definitions, and additional items reflecting the WHO standards for improving maternal and newborn care [27,28], informed by Stierman *et al.* [29]. Our labour and delivery readiness index included a total of 55 items covering the following five domains: equipment, medicines and commodities, basic amenities, human resources and guidelines, and performance of basic emergency obstetric and newborn care (BEmONC) signal functions in the last 12 months (Table S1 in the **Online Supplementary Document**).

For SSNC readiness, we generated a weighted additive readiness score whereby each domain (rather than each item) was weighted equally towards the overall score. This was deemed more appropriate than a simple additive method given differing number of items per domain. We adapted the SSNC readiness index developed by Sheffel *et al.* [30] based on their mapping and extraction of the 2020 WHO Standards for improving the quality of small and sick newborns care [31], Survive and Thrive [32], and works by Moxon *et al.* [33]. The SSNC index included a total of 50 items covering the following eight intervention domains, as well as a general facility readiness domain: immediate newborn care and routine care (including newborn drying, immunisation, hygienic cord care, vitamin K, and eye care), early initiation and support for breastfeeding, neonatal resuscitation, prevention of mother-to-child transmission of HIV, Kangaroo Mother Care (KMC), detection and management of neonatal infection, comfort and pain management, and detection and management of hypoglycaemia (Table S2 in the **Online Supplementary Document**). Facilities that did not offer one or more of the above interventions received a value of 0 for all the corresponding items within the intervention domain.

#### Person-centred maternity care

We estimated PCMC scores following Afulani *et al*.’s methodology, whereby a numeric value ranging from 0 to 3 is assigned to each of the 30 items making up the PCMC scale [20,21] (Table S3 in the **Online Supplementary Document**). Responses are on a 4-point scale (no never, yes a few times, yes most of the time, yes all the time) and the highest value of 3 is attributed to the response option reflecting optimal person-centred behaviour. Values for all 30 items are summed for a maximum score of 90 points for the full PCMC scale; a higher score therefore reflects higher PCMC. We also generated domain-specific scores for each of the three PCMC subscales: dignity and respect (6 items for a total of 18 points), communication and autonomy (9 items for a total of 27 points), and supportive care (15 items for a total of 45 points) [21].

### Statistical analysis

#### Maternal and newborn health service readiness

The analysis of service readiness was restricted to facilities offering labour and delivery care. We computed overall and domain-specific readiness scores for labour and delivery care as well as SSNC in each city. These were measured on a continuous scale ranging from 0 to 1 and were rescaled to a 100 for improved interpretability as a percentage. We evaluated floor and ceiling effects by reporting the proportion of facilities with very low (0–5%) or very high (95–100%) readiness scores: these were found to be below 10% for both service areas, indicating adequate capacity to discriminate between facilities with very low/very high readiness. Additionally, we disaggregated readiness scores by facility type and managing authority. We used the same items to define service readiness in hospitals and lower-level facilities, reflecting the same standards for high quality care. However, given that some items such as infant incubators, resuscitation tables, x-ray machines and morphine were not expected to always be available in lower-level facilities, we also calculated the maximum score expected in lower-level facilities when excluding such items.

In Lusaka and Ouagadougou, we compared mean readiness scores between facilities serving and not serving informal settlements: we assessed *t* test assumptions, and based on Levene’s test for equal variance, we ran a *t* test with the appropriate variance assumption. We also compared the proportion of facilities with low (<60%), moderate (60%-80%) and high (>80%) readiness scores; an arbitrary threshold of 80% was used to reflect facilities with most items available and functional.

#### Association between facility readiness and experience of person-centred maternity care

We conducted an exact match linking of individual women’s self-reported PCMC scores and the facilities’ labour and delivery care readiness scores, for each city separately. We linked the two separate data sources (the client exit survey and the health facility assessment) by facility, and we assigned to each woman’s self-reported PCMC score (from the client exit survey), the labour and delivery readiness score of the facility in which she gave birth (from the health facility assessment). The proportion of surveyed women linked to a facility was 97.3% in Lusaka, 99.3% in Nairobi and 100% in Ouagadougou. A subset of facilities were included in the linking analysis, given client exit surveys were conducted in select informal settlements.

For each city, we ran a multilevel random intercept linear regression model of the PCMC score (out of 90) on the overall rescaled labour and delivery readiness score (%). In this multilevel model, level-1 referred to individual women and level-2 referred to the facility they attended, thus accounting for clustering of women within facilities and variability in PCMC scores across facilities. We ran crude and adjusted models, controlling for structural factors (women’s education and employment), intermediary factors (women’s age, marital status, parity, pregnancy complications, history of miscarriage/stillbirth, antenatal care) and health systems factors (facility type and managing authority, provider type assisting at birth, length of facility stay, receipt of maternal and newborn postnatal care and content of care prior to discharge). In the final model, we included the covariates that had bivariate associations with PCMC significant at *P*-value <0.2, and we assessed multicollinearity using variance inflation factors. As supplementary analyses, we tested the association between labour and delivery readiness scores and each PCMC domain separately.

## RESULTS

### Characteristics of facilities offering maternal and newborn health services

In Lusaka, 80.7% of facilities serving informal settlements were public health centres and 12.9% were public hospitals, whereas those not serving informal settlements were mostly private facilities. In Nairobi and Ouagadougou, 92% and 85.3% of facilities serving informal settlement respectively were health centres, the majority being private. Most facilities across cities had continuous power supply and access to an improved water source, however less than half had an ambulance or a vehicle available 24 hours on the day of the survey (**Table 1**).

**Table 1:** Characteristics of facilities offering maternal and newborn health services by city

	Lusaka		Nairobi	Ouagadougou	
	**Serving informal settlement (n = 31)**	**Not serving informal settlements (n = 88)**	**Serving informal settlements (n = 38)**	**Serving informal settlements (n = 115)**	**Not serving informal settlements (n = 215)**
Facility type and managing authority*, %					
Public health centre/other	80.7	30.7	21.0	27.0	26.5
Private for-profit health centre/other	6.4	45.5	50.0	40.9	32.6
Private non-profit health centre/other	0.0	13.6	21.0	17.4	7.4
Public hospital	12.9	4.5	7.9	1.7	6.5
Private for-profit hospital	0.0	5.7	0.0	6.9	19.5
Private non-profit hospital	0.0	0.0	0.0	6.1	7.4
**Staffing (min–max range)**					
Mean number of nurses/midwives per health centre	25.4 (1–107)	12.7 (1–65)	8.7 (0–50)	4.2 (0–24)	2.9 (0–38)
Mean number of nurses/midwives per hospital	164.5 (90–287)	75.2 (2–296)	69.7 (28–140)	14.1 (0–173)	9.7 (0–177)
Mean number of physicians/obstetricians per health centre	45.1 (0–190)	19.6 (0–141)	0.2 (0–2)	2.1 (0–22)	6.7 (0–52)
Mean number of physicians/obstetricians per hospital	310 (18–619)	70.0 (1–236)	38.7 (1–68)	15.3 (0–59)	21.7 (0–128)
**Number of beds (min–max range)**					
Mean number of beds (inpatient, observation and maternity, excluding delivery beds) per health centre	5.1 (0–40)	7.7 (0–65)	5.6 (0–30)	8.7 (0–52)	6.6 (0–29)
Mean number of beds (inpatient, observation and maternity, excluding delivery beds) per hospital	91.0 (17–205)	18.0 (4–50)	72.7 (0–200)	14.4 (2–48)	20.8 (0–207)
**Basic amenities, %**					
Continuous power supply (last 7 days)	74.2	77.3	65.8	66.1	65.1
Access to improved water source	87.1	90.9	84.2	85.2	84.7
**Emergency transport, %**					
Functional 24h ambulance or vehicle with driver	32.3	48.9	31.6	21.7	26.5
**Laboratory & pharmacy capacity, %**					
Availability of in-house laboratory for diagnostic testing	51.6	68.2	94.7	74.8	76.3
Availability of in-house storage of pharmaceuticals	100.0	98.9	97.4	85.2	73.5

*Facility types are defined as follows: in Lusaka health centre/other refers to urban health posts, health centres or clinics, and hospitals refer to first and second level hospitals. In Nairobi, health centre/other includes facility levels 2 & 3, hospital includes health facility levels 4 & 5. In Ouagadougou health centre/other includes ‘niveau 1 échelon 1’ facilities (cabinet médical, centre de santé et promotion sociale, cabinet de soins infirmiers, clinique d'accouchement, dispensaire isolée, infirmerie), hospital refers to ‘niveau 1 échelon 2’ facilities (Centre médical avec antenne chirurgicale, clinique, centre médical).

Approximately one third (35.5%) of facilities offering MNH services in Lusaka’s informal settlements offered labour and delivery care; In Nairobi and Ouagadougou’s informal settlements, close to half did. The reported availability of basic emergency obstetric and newborn care (BEmONC) varied between 32.3% to 47.4% in informal settlements across cities, however less than 16% of facilities reported offering all seven BEmONC signal functions. Only 5.2% and 9.3% of facilities serving and not serving informal settlements respectively in Ouagadougou administered oxygen. KMC was available in less than 20% of facilities, with lower availability in informal settlements: in Ouagadougou’s informal settlements, only 8.7% of facilities offered KMC (Table S4 in the **Online Supplementary Document**).

### Facility readiness for labour and delivery care

**Figure 1:**
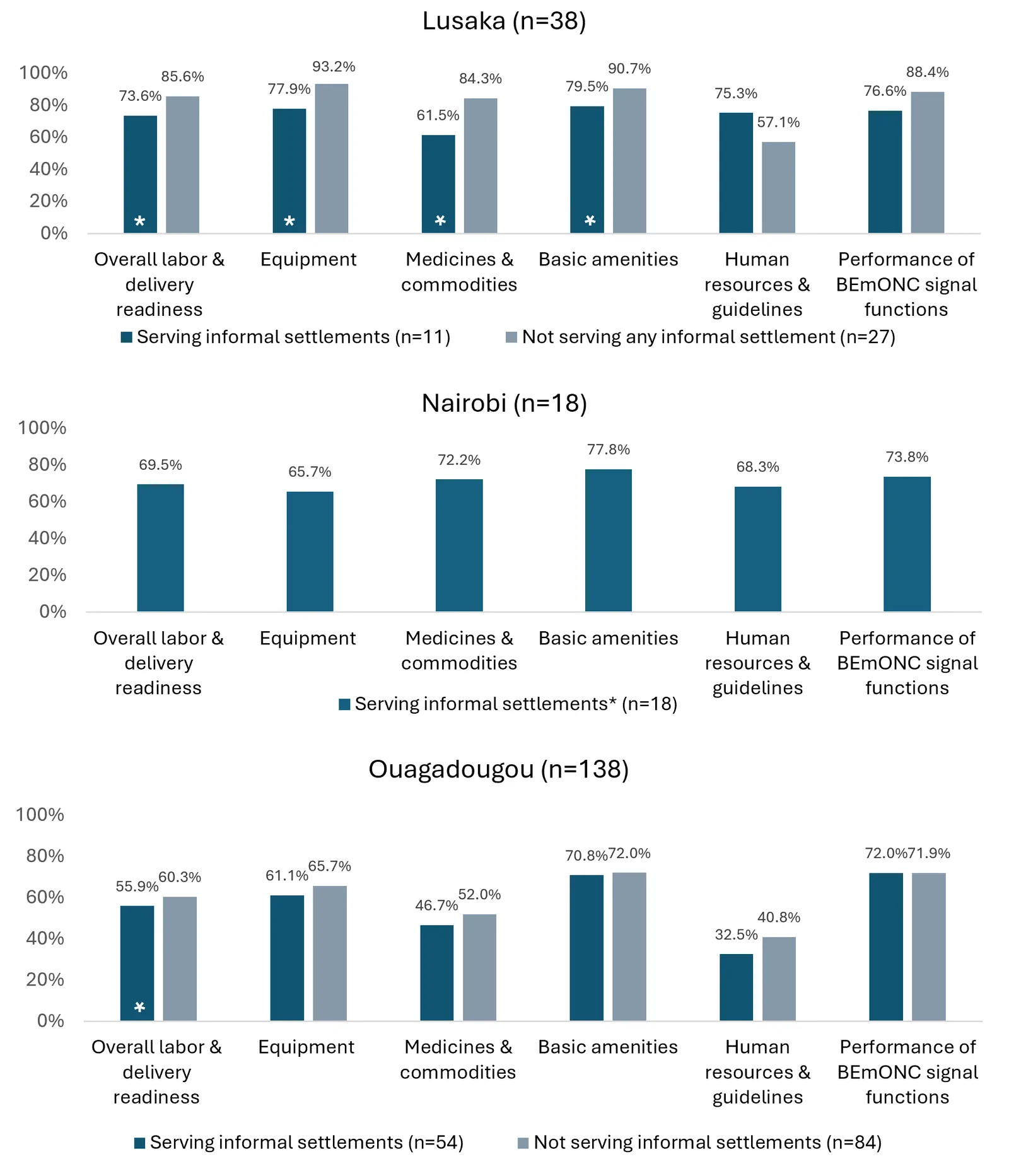
Labour and delivery care readiness scores (%) by domain and city. *Difference in mean readiness score between facilities serving and not serving informal settlements significant at *P* < 0.05 based on *t* test for the following domains: Lusaka: overall labour and delivery readiness, equipment, medicines and commodities, basic amenities; Ouagadougou: overall labour and delivery readiness.

**Figure 2:**
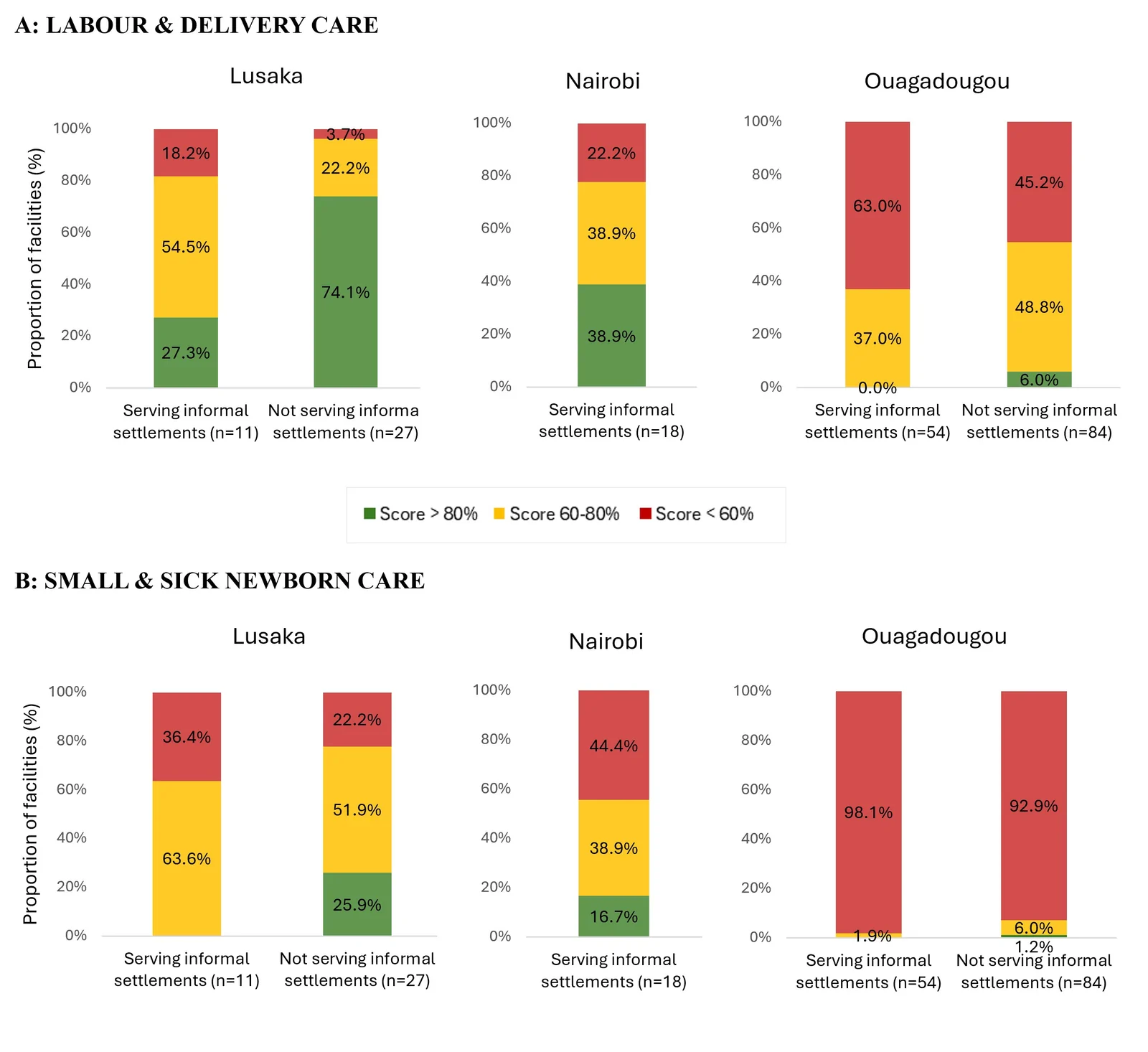
Proportion of facilities with high, moderate and low readiness scores for labour and delivery care (Panel A) and small and sick newborn care (Panel B).

The overall mean readiness score for labour and delivery care in facilities serving informal settlements was 73.6% (standard deviation (SD) = 12.5) in Lusaka, 69.5% (SD = 19.4) in Nairobi and 55.9% (SD = 11.9) in Ouagadougou. It was statistically significantly higher in facilities not serving informal settlements in Lusaka and Ouagadougou at 85.6% (SD = 9.2) and 60.3% (SD = 12.7), respectively (**Figure 1**; Figure S1 and Table S5 in the **Online Supplementary Document**). Similarly, in both these cities, there was a higher proportion of facilities serving informal settlements with low (<60%) readiness scores, and a smaller proportion with high readiness scores (>80%). For instance, only 27.3% of facilities serving informal settlements in Lusaka had high readiness compared to 74.1% of facilities not serving informal settlements. In Ouagadougou, no facility serving informal settlements had a readiness score above 80% (**Figure 2****,** Panel A).

Facilities serving informal settlements in Lusaka had statistically significantly poorer readiness scores for all domains except human resources and performance of BEmONC signal functions, and were least ready in terms of medicines and commodities with a domain score of 61.5% (**Figure 1**; Table S5 in the **Online Supplementary Document**). Key items that were lacking in Lusaka’s informal settlements included injectable vitamin K, antihypertensive drugs, calcium gluconate and oxygen, in addition to basic equipment items (examination lights, vacuum aspirators and clean towels to dry newborns) (Figure S2, Panel A and Table S6 in the **Online Supplementary Document**). In Ouagadougou, both facilities serving and not serving informal settlements were deficient in the availability of trained human resources and guidelines, with domain scores of 32.5% (SD = 25.0) and 40.8% (SD = 26.4) respectively (**Figure 1**; Table S5 in the **Online Supplementary Document**). Checklists for essential childbirth care, staff trained in all areas of labour and delivery care, oxygen, misoprostol, and infant incubators were particularly unavailable in informal settlement facilities (Figure S2, Panel C; Table S6 in the **Online Supplementary Document**). In Nairobi’s informal settlements, domain scores ranged from 65.7% (SD = 23.8) for equipment to 77.8% (SD = 19.0) for basic amenities (**Figure 1**; Table S5 in the **Online Supplementary Document**), though the following items generally had poor availability: vacuum aspirator, infant incubator, neonatal resuscitation bag and mask, and guidelines/checklists (Figure S2, Panel B; Table S6 in the **Online Supplementary Document**).

When disaggregating labour and delivery readiness scores by facility type and managing authority, public hospitals serving informal settlements had higher readiness than their counterparts not serving informal settlements. In Lusaka, the four public hospitals in informal settlements had a higher overall readiness at 86.6% compared to 77.8% in the three public hospitals not serving informal settlements. In Ouagadougou, private facilities serving informal settlements, representing the majority of facilities serving these areas, were the least ready for labour and delivery care. Similarly, in Nairobi, private health centres received the lowest score of 62.6% and public hospitals received the highest score of 87.7%. Across cities, none of the facilities serving informal settlements, except one public hospital in Lusaka, met the maximum readiness score expected in lower-level facilities (Figure S3 and Table S7 in the **Online Supplementary Document**).

### Facility readiness for small and sick newborn care

**Figure 3:**
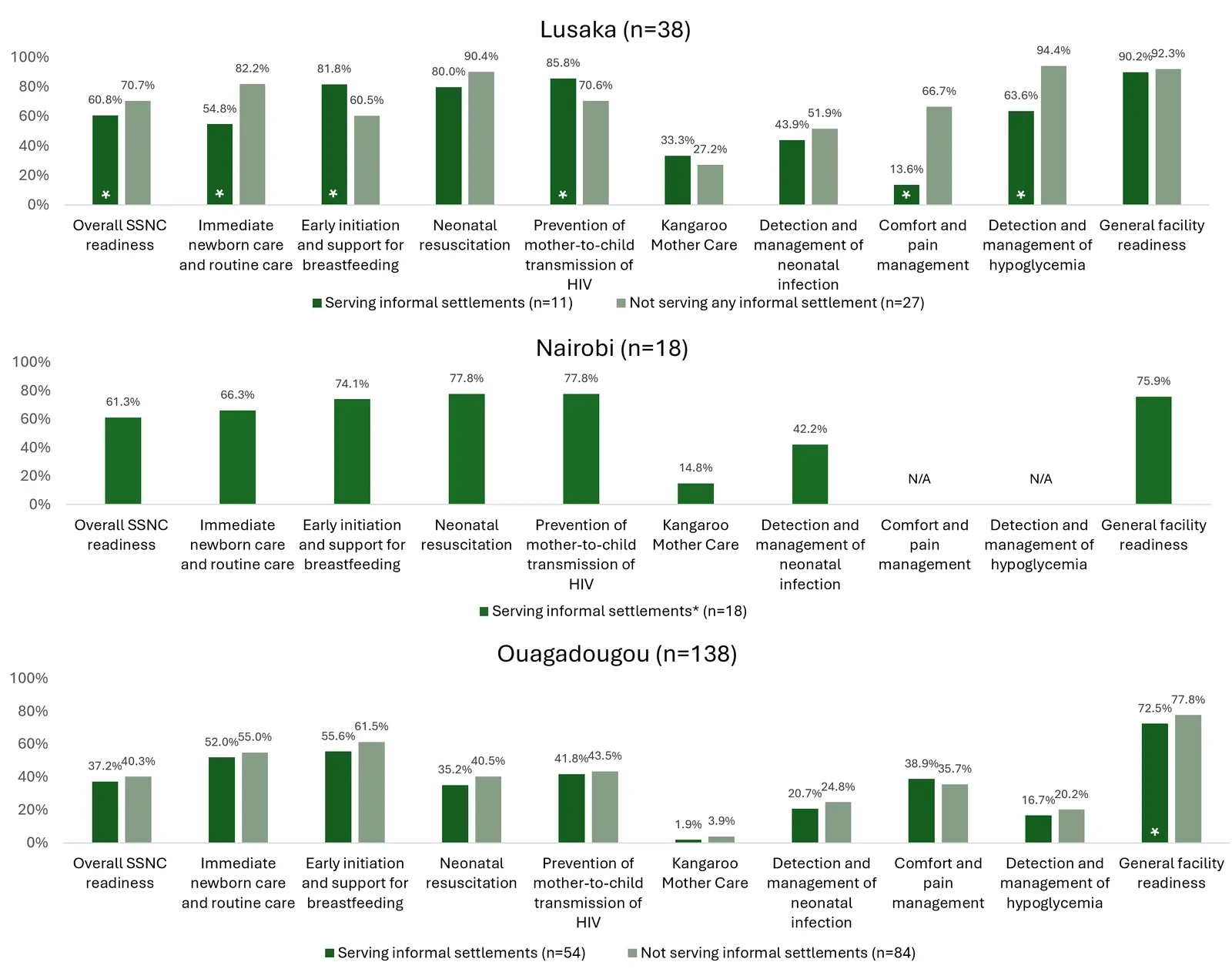
Small and sick newborn care readiness scores (%) by domain and city. *Difference in mean readiness score between facilities serving and not serving informal settlements significant at *P* < 0.05 based on *t* test for the following domains: Lusaka: overall SSNC readiness, immediate newborn care and routine care, early initiation and support for breastfeeding, prevention of mother-to-child transmission of HIV, comfort and pain management, detection and management of hypoglycemia; Ouagadougou: general facility readiness.

The overall mean SSNC readiness score in facilities serving informal settlements was 60.8% (SD = 14.5) in Lusaka, 61.3% (SD = 17.9) in Nairobi and much lower at 37.2% (SD = 9.3) in Ouagadougou. In Lusaka, it was statistically significantly higher at 70.7% (SD = 11.4) in facilities not serving informal settlements (**Figure 3**; Table S5 and Figure S4 in the **Online Supplementary Document**). In Ouagadougou, 98.1% and 92.9% of facilities serving and not serving informal settlements respectively had low readiness for SSNC (scores <60%), and no facility serving informal settlements scored over 80%. The latter was also observed in Lusaka, whereas 25.9% of facilities outside informal settlements scored over 80% (**Figure 2****,** Panel B).

In Lusaka’s informal settlements, SSNC intervention domain scores ranged from 13.6% for comfort and pain management to 85.8% for PMTCT. KMC readiness was consistently poor across areas, with large variability across facilities. This was also found in Nairobi’s informal settlements where the KMC domain score was 14.8% (SD = 29.1). In Ouagadougou, readiness for SSNC was generally poorer than in Lusaka and Nairobi. Intervention domain scores in informal settlements ranged from a meager 1.9% (SD = 6.6) for KMC to 55.6% (SD = 25.9) for early initiation of breastfeeding (**Figure 3**; Table S5 in the **Online Supplementary Document**). Further analyses pointed to the general unavailability of trained staff in KMC, as well as guidelines and registers for KMC and neonatal sepsis across all facilities, both serving and not serving informal settlements (Figure S5, Panels A–C & Table S8 in the **Online Supplementary Document**).

Public hospitals serving informal settlements consistently had higher SSNC readiness than both public and private hospitals not serving informal settlements in Lusaka and Ouagadougou. In all cities, none of the facilities serving informal settlements achieved the maximum readiness score expected in lower-level facilities (Figure S6 and Table S7 in the **Online Supplementary Document**).

### Association between labour and delivery readiness and PCMC

In Lusaka, 424 women were linked to 10 facilities, in Nairobi 411 women were linked to 16 facilities, and in Ouagadougou 401 women were linked to 54 facilities. Most women were aged 20–34 years old, were unemployed, and were attended by a nurse or midwife during childbirth. In Lusaka and Nairobi, the majority of women gave birth in public hospitals, whereas in Ouagadougou they gave birth in public health centres (Tables S9 and S10 in the **Online Supplementary Document**).

Multilevel models adjusting for relevant structural, intermediary and health systems determinants of PCMC suggested no statistically significant effect of giving birth in a facility with higher readiness on women’s overall PCMC experience (**Table 2**). As a sensitivity analysis, we re-ran this model on a logit-transformed PCMC outcome, and the results were consistent. Our supplementary analyses for each PCMC domain separately indicated that, while statistically significant associations were observed, the magnitudes remained small: In Nairobi, a 10-point increase in facility readiness was associated with a 0.6-point decreased communication and autonomy score (out of 27 points), and a 0.8-point increased supportive care score (out of 45 points). In Lusaka, it was associated with a 2.2-point decreased supportive care score (Table S11 in the **Online Supplementary Document**).

**Table 2:** Associations between facilities’ labour and delivery care readiness scores (%) and women’s PCMC scores (out of 90 points) in study areas by city

	Crude model			Adjusted Model		
	Coefficient (95% CI)*	***P*-value**	**n**	Coefficient (95% CI)*	***P*-value**	**n**
Lusaka†	−0.14 (−0.42, 0.14)	0.317	424	−0.12 (−0.36, 0.11)	0.301	411
Nairobi‡	−0.04 (−0.25, 0.18)	0.725	411	0.04 (−0.10, 0.17)	0.579	406
Ouagadougou§	0.05 (−0.12, 0.22)	0.580	401	0.06 (−0.11, 0.23)	0.470	373

*Coefficient interpretation: average change in PCMC score out of 90 points associated with attending a facility that had a 1 percentage point higher labour and delivery readiness score

†Lusaka model adjusted by 7 factors: women’s employment, type of provider assisting during delivery, maternal PNC check received before discharge, PNC counselling on danger signs received before discharge, PNC counseling on family planning received before discharge, blood pressure checked before discharge, newborn PNC check received before discharge.

‡Nairobi model adjusted by 10 factors: delivery facility type & managing authority, women’s age, women’s marital status, type of provider assisting during delivery, maternal PNC check received before discharge, PNC counseling on danger signs received before discharge, PNC counselling on family planning received before discharge, blood pressure checked before discharge, newborn PNC check received before discharge, appointment received for next PNC check before discharge.

§Ouagadougou model adjusted by 11 factors: delivery facility type & managing authority, women’s employment, women’s age, ANC4+, type of provider assisting during delivery, maternal PNC check received before discharge, PNC counselling on danger signs received before discharge, PNC counselling on family planning received before discharge, blood pressure checked before discharge, newborn PNC check received before discharge, appointment received for next PNC check before discharge.

## DISCUSSION

Our study in facilities serving informal settlements in Nairobi, Lusaka and Ouagadougou revealed sub-standard readiness for labour and delivery care, and even poorer readiness for SSNC. Readiness scores in informal settlement facilities ranged from 55.9% in Ouagadougou to 73.6% in Lusaka for labour and delivery care, and from 37.2% in Ouagadougou to 61.3% in Nairobi for SSNC. Importantly, we found that facilities serving informal settlements were statistically significantly less ready than those not serving informal settlements, although key items were lacking across all facilities. Our exact match linking analysis did not detect a statistically significant effect of higher labour and delivery readiness on women’s overall experience of PCMC.

Other studies have assessed readiness for MNH services in sub-Saharan Africa [27,34–36]: one study measuring childbirth care readiness in Ethiopia reported median scores ranging from 75% to 96% [27]; another one using HHFA data in Malawi, Mozambique and Tanzania estimated SSNC readiness scores ranging from 53.7% to 80.7% [37]. A recent analysis in Burkina Faso highlighted a declining trend in the availability and readiness for emergency obstetric and newborn care between 2014 and 2018, driven by insufficient trained staff and medicines. Authors suggested this may be linked to rising insecurity and the increased demand for health services since the adoption of free maternal healthcare in 2016 [36]. While differences in readiness item selection and definition make comparisons across studies difficult, consistent with our findings, these studies also reported higher facility readiness among hospitals compared to health centres [27,36].

Despite targeted efforts, substantial gaps in readiness for critical maternal and newborn care interventions persist across settings, and particularly in provider training. In our study, less than 8% of facilities (both serving and not serving informal settlements) in Ouagadougou had staff trained in KMC in the previous 2 years; it was less than 30% for newborn resuscitation. In line with our findings, studies from Ethiopia and Northeastern Nigeria have highlighted health worker training gaps in the management of eclampsia and newborn resuscitation [38,39]. Importantly, there has been a lack of measures assessing provider knowledge and competency as health facility assessments do not measure the training content nor assess providers’ knowledge, posing an additional measurement constraint [29].

KMC services offer the potential to save lives of preterm and low birthweight (LBW) babies [40] for whom skin-to-skin contact offers an inexpensive alternative to infant incubators, particularly in low-resource settings [41,42], however substantial gaps persist. We found poor availability and readiness for KMC services across facilities serving and not serving informal settlements, especially in Ouagadougou. A systematic review of KMC implementation in SSA identified health systems barriers linked to insufficient space to carry out KMC, as well as inadequate staff training and guidelines [43]. This is in line with our results and others’ [37]: in Ouagadougou, we found that zero and 2.4% of facilities serving and not serving informal settlements respectively had a dedicated location for the caregiver to provide KMC overnight. Similarly, no facility serving informal settlements in Nairobi and Ouagadougou had KMC caps/hats for newborns. One of the factors explaining this result is that very few facilities offer KMC in Ouagadougou, and most newborns requiring KMC are referred to higher-level facilities, although there is no formal policy restricting the service to these facilities. Strong leadership prioritising KMC implementation, readiness and provider training, especially in lower-level facilities serving vulnerable populations, are critical to improve both access to and quality of SSNC, and ensure newborn survival [43].

Addressing disparities in service readiness between facility types and managing authorities is essential in informal settlements where lower-level private facilities often lack adequate resources and regulation. Our analysis highlighted such differences: service readiness was higher in public hospitals serving informal settlements compared to those not serving informal settlements, and lowest in private facilities serving informal settlements, contrary to previous findings [44]. High delivery volumes in informal settlements may lead to more resources allocated to public hospitals in these areas, though further investigation is needed. Our findings also reflect the sprawling of unregulated, poorly equipped informal private facilities in low-income urban neighborhoods in SSA [45,46]. Efforts are needed to ensure that private facilities serving informal settlements are adequately regulated and ready to offer essential services. Importantly, the lower readiness in facilities serving informal settlement also reflects the combination of facility types in these areas, with fewer hospitals and more health centres, compared to facilities not serving informal settlements. For instance, in Ouagadougou, only two public hospitals served informal settlements, compared to ten outside informal settlements.

Consistent with our findings, several studies have reported higher readiness for MNH services in higher-level facilities [29,47,48]. An analysis in five African countries found that primary level facilities, which accounted for more than 90% of facilities offering obstetric services, had very low quality of maternal care based on readiness, referral systems and routine emergency care [44]. The latest DHS suggest that in the Centre region of Burkina Faso, over 65% of births occurred in lower-level facilities (outside hospitals) [49], which also made up the majority of facilities serving informal settlements in our study, whereas in Nairobi county and Lusaka province, most births occurred in hospitals [50,51]. Quality improvement efforts are therefore critical in facilities where the majority of births occur.

While we hypothesised that facilities with better structural quality would offer more competent, empowered and motivated staff, and this would translate into a better experience of maternity care, our findings did not support this. Other studies have reported significant but small magnitudes for the effect of structural quality on process quality of antenatal and sick child care in LMICs [52,53]. Most components of PCMC refer to women’s interactions with health providers, and their own perspectives on structural aspects such as the cleanliness, crowdedness and safety of the facility. These dimensions of PCMC are not directly captured in facility readiness indices, and similarly, the PCMC scale does not directly capture elements of facility readiness such as the availability of equipment and supplies, potentially explaining the lack of effect found. Importantly, facility readiness does not reflect health providers’ workload, stress/burnout, implicit bias and motivations, which have a large role in the provision of PCMC [54–56]. In line with our previous findings of poorer communication experiences in hospitals (compared to health centres) [24], our supplementary analyses identified a small but significant negative effect of higher facility readiness on women’s communication and autonomy scores in Nairobi’s urban informal settlements. This reinforces the result that higher structural quality does not necessarily translate into health providers’ ability to offer person-centred care. Further research is needed to understand the complex associations between facility readiness, provision of care, and patient experiences of care, as well as health providers’ barriers and enablers of PCMC in the context of urban informal settlements.

Our study has several strengths: to our knowledge, it is the first study comparing service readiness between facilities serving and not serving urban informal settlements, in addition to a multi-country comparison. The exact match linking between women’s client exit surveys and health facility assessments allowed for triangulation across data sources. Our results offer actionable insights for country-specific targeted interventions addressing the unavailability of key items needed for quality care. There are also limitations worth mentioning: facility readiness scores for labour and delivery and SSNC are influenced by the items that define them, and are not standardised across studies; item selection in our study was based on the existing literature and WHO guidelines, and was identical for facilities serving and not serving informal settlements. PCMC scores relied on women’s self-report and were subject to recall bias; this was minimised by limiting the recall period to 2 weeks following facility discharge. Specific components of the PCMC scale are inherently subjective, and poor provider behaviour may be normalised in low-resource settings. In Lusaka and Ouagadougou, the client exit survey was conducted within 2 to 3 years of the health facility assessment. These temporal differences may affect the associations we tested and limit comparisons across cities, although facility readiness measures are unlikely to vary substantially within this time frame. Differences in definitions of urban informal settlements across cities, and the unavailability of service readiness data beyond informal settlements in Nairobi may further affect comparability across settings; our findings should be interpreted in light of these differences.

## CONCLUSIONS

In conclusion, our study brings to light sub-standard service readiness for labour and delivery care and SSNC across facilities in Nairobi, Lusaka and Ouagadougou. Those serving urban informal settlements were particularly disadvantaged. While we found no significant association between labour and delivery care readiness and women’s overall experience of PCMC, further research is needed to better understand the relationship between structural quality, process quality, and experience of care. Despite nearly universal institutional deliveries in sub-Saharan African cities, the road to meeting the Every Newborn Action Plan and Sustainable Development Goal targets for maternal and newborn health is still long. Health systems investments are warranted to improve the availability and functionality of key items, as well as ensure staff are trained in critical service areas, across facility types and both public and private sectors. Quality of care for essential MNH services remains inadequate for marginalised urban populations, and it is imperative to ensure that facilities serving these populations have the structural quality in place, both in terms of physical and human resources, to drive improvements in maternal and newborn survival.

## Additional material


Online Supplementary Document

## Data Availability

**Data availability:** The data sets generated and/or analysed during the current study can be made available from the corresponding author on reasonable request.
