# Supplementary material for: Hidden cities, hidden gaps: measuring facility readiness for maternal and newborn health services and its association with person-centred maternity care in sub-Saharan Africa’s urban informal settlements
Source: J Glob Health. 2026 Sep 25;16:04343. doi: 10.7189/jogh.16.04343 (PMC13613106; doi:10.7189/jogh.16.04343)
Supplement: Supplementary file 1 [file jogh-16-04343-s001.pdf]

Supplement to: Jiwani SS, Mutua M, Cisse K, Jacobs C, Njeri A, Musukuma M, Adero G, Sheffel A, Munos MK, Faye C, Boerma T, Amouzou A. Hidden cities, hidden gaps: measuring facility readiness for maternal and newborn health services and its association with person-centred maternity care in sub-Saharan Africa's urban informal settlements. J Glob Health. 2026;16:04343.

Table S1. Definitions of items included in labor and delivery readiness index.

Domain	Item description	Lusaka	Nairobi	Ouagadougou
Equipment	Blank partograph: observed available and reported functionality in service site	x	x	x
	Examination light: Functioning spotlight source that can be used for patient examinations; a functional flashlight is accepted. Observed available and reported functionality in service site.	x	x	x
	Blood pressure apparatus: Digital BP machine or manual sphygmomanometer with stethoscope. Observed available and reported functionality in service site.	x	x	x
	Foetal stethoscope: Foetal stethoscope/pinard/ foetoscope/digital doplar. Observed and functional in service site.	x	x	x
	Delivery bed: delivery bed w/stirrups. Observed available and reported functional in service site.	x	x	x
	Delivery pack (or cord clamp, episiotomy scissors, scissors/blade to cut cord, suture material with needle, and needle holder). Observed available and reported functionality in service site.	x	x	x
	Vacuum aspirator or D&C kit and speculum. Observed available and reported functionality in service area.	x	x	x
	Manual vacuum extractor or forceps for outlet application. Observed available and reported functionality in service area.	x	x	x
	Sterilization equipment: dry heat sterilizer or an autoclave; if the machine is not electric, the heat source needs to be available and (If relevant) functioning (e.g., wood or gas is present for the autoclave). Observed available and reported functional anywhere in the facility.	x	x	x

	Resuscitation table for newborn with heat source. Observed available and reported functional in service area.	x	x	x
	Infant incubator. Observed available and reported functional anywhere in facility.	x	x	x
	Suction pump and catheter or suction bulb for newborn. Observed available and reported functional in service area.	x	x	x
	Neonatal resuscitation bag and mask (size 1 & size 0). Observed available and reported functionality in service site.	x	x	x
	Adult resuscitation bag and mask. Observed available and reported functionality in service site.	x	x	x
	Infant weighing scale with 100g gradation. Observed available and reported functionality in service site.	x	x	x
	Pulse oximeter. Observed available and reported functionality in service site.	x	x	x
	Thermometer: Manual, electronic or digital. Observed available and reported functionality in service site.	x	x	x
	Latex gloves: If equivalent non latex gloves are available this is acceptable; Either sterile or non sterile; latex or equivalent. Observed available in service site.	x	x	x
	Hand hygiene: soap and water, or alcohol based rub. Observed in service site.	x	x	x
	Clean towel for drying newborn. Observed available in service site.	x	x	x
	Non-sharps waste container: Waste receptacle bin with lid and plastic bin liner for non-sharps infectious waste. Observed available in service site.	x	x	x
	Sharps container. Observed available in service site.	x	x	x
	Environmental disinfectant. Observed available in service site.	x	x	x
Medicines & commodities	Injectable uterotonic: Oxytocin (or country-specific equivalent) observed and stored in cold storage, at least one not expired in service site or where routinely stored.	x	x	x
	Injectable betamethasone or dexamethasone. Observed at least one not expired in service site or where routinely stored.	x	x	x
	IV solution and perfusion kit: Intravenous solution for infusing medicines (Ringers lactate, Normal saline, or D5W) and infusion set. Observed available and at least one not expired in service site or where routinely stored.	x	x	x

	Misoprostol tablet 200 mcg. Observed at least one not expired in service site or where routinely stored.	x	x	x
	Oxygen and administration equipment: Oxygen currently in unit or oxygen currently not in unit, but report that unit calls centrally for O2 if needed and humidifier, flowmeter, oxygen delivery apparatus are observed available and functioning in service site.	x	x	x
	Antibiotic eye ointment: Eye cream for newborn or for trachoma (tetracycline or country-specific equivalent). Observed at least one not expired where routinely stored.	x	x	x
	Injectable magnesium sulphate, 50% strength or alternative. Observed and not expired in service site or where routinely stored.	x	x	x
	Injectable antibiotic for maternal/neonatal sepsis: Broad-spectrum injectable antibiotic treatment of sepsis in mother and newborn. Specific combination- (Ampicillin powder for injection + gentamicin injection) or (penicillin injection + gentamicin injection) or ceftriaxone injection. Observed at least one not expired in service site or where routinely stored.	x	x	x
	Skin disinfectant for cord care: 4% chlorhexidine.* Observed available and at least one not expired in service site or where routinely stored.		x	x
	Injectable Vitamin K. observed available and not expired in service site or where routinely stored.	x	x	x
	Injectable calcium gluconate. observed available and not expired in service site or where routinely stored.	x	x	x
	Antihypertensive (hydralazine, nifedipine, or methyldopa). Observed at least one not expired in service site or where routinely stored.	x	x	x
	Injectable metronidazole. Observed at least one not expired in service site or where routinely stored.	x	x	x
	Injectable diazepam. Observed at least one not expired in service site or where routinely stored.	x		x
Human resources & guidelines	At least one staff in service area trained in newborn resuscitation using bag and mask in last 2 years.	x	x	x
	At least one staff in service area trained in essential childbirth care in the last two years.	x	x	x

	At least one staff in service area trained in essential newborn care in last 2 years.	x	x	x
	guidelines for essential childbirth care observed in service site.	x	x	x
	checklist or job aids for essential childbirth care observed in service site.	x	x	x
	guidelines for essential newborn care observed in service site.	x	x	x
	skilled attendant present on call (onsite or in near proximity) 24 hours.	x	x	x
Basic amenities	Improved water source: Main water source located on premises and from which water is available. Improved water sources include: piped, public tap, standpipe, tubewell/borehole, protected dug well, protected spring, rain water. If public tap, standpipe, tubewell/borehole, protected dug well, protected spring, rain water.	x	x	x
	Improved sanitation facility: Flush/pour flush to piped sewer system or septic tank, pit latrine with slab, or composting toilet available in service site or oupatient area.	x	x	x
	Electricity available at all times when facility was open in last 7 days and/or back-up energy.	x	x	x
	Emergency transport (available on day of survey): Facility has access to a functional ambulance or other vehicle for emergency transportation for clients that is either stationed at this facility or that the facility can call for, and vehicle available and functional today.	x	x	x
Performance of BEmONC signal functions in last 12 months	Parenteral administration of antibiotics (IV or IM) for mothers	x	x	x
	Parenteral administration of anticonvulsants (magnesium sulfate) for management of pre-eclampsia and eclampsia (IV or IM)	x	x	x
	Parenteral administration of oxytocic for treatment of postpartum hemorrhage (IV or IM)	x	x	x
	Removal of retained products of conception using D&C or manual vacuum aspiration	x	x	x
	Assisted vaginal delivery using manual vacuum extraction (MVE) or forceps	x	x	x
	Manual removal of placenta	x	x	x
	Neonatal resuscitation with bag and mask	x	x	x

*Based on country-specific recommendations: chlorhexidine for cleaning cord excluded in Lusaka as it is not a national policy.

Table S2. Definitions of items included in small & sick newborn care readiness index.

Domain	item description	Lusaka	Nairobi	Ouagadougou
**Immediate newborn care and routine care**	Clean towel for drying newborn. Observed available in service site.	x	x	x
	Cord cutting supplies: sterile scissors and/or sterile blade to cut cord AND umbilical cord clamp (sterile ligatures or clamp of Barr) OR delivery pack. Observed available and functional in service site.	x	x	x
	Radiant heater/warmth source or infant incubator. Observed available and reported functionality anywhere in the facility.	x	x	x
	Injectable Vitamin K. observed available and not expired in service site or where routinely stored.	x	x	x
	Antibiotic eye ointment: Eye cream for newborn or for trachoma (tetracycline or country-specific equivalent). Observed at least one not expired where routinely stored.	x	x	x
	Skin disinfectant for cord care: 4% chlorhexidine.* Observed available and at least one not expired in service site or where routinely stored.		x	x
	Immunization supplies: 1) BCG, 2) Hep B, and 3) OPV observed in pharmacy or anywhere in the facility where medicines are routinely stored; at least one with valid expiration date observed available. 4) Single use needles and syringes are observed available in the delivery service provision area, OPD, pharmacy, or immunization service area.<br>5) Guidelines containing information on child immunization or the national immunization schedule are observed available in the immunization service provision area.<br>6) A refrigerator dedicated for vaccine storage with a temperature monitoring device and consistent electricity supply is available in the facility OR the facility has a cold box/vaccine carrier with ice packs in the immunization service area. Calculated as proportion of items available out of 6.	x	x	x
**Early initiation and support for breastfeeding**	Guidelines for breastfeeding and promotion of breastfeeding. Observed available in service site.	x	x	x
	At least one staff in service site trained in breastfeeding and promotion of breastfeeding in last two years.	x	x	x

	Facility routinely practices breastfeeding initiation within 1 hour of birth.	x	x	x
**Neonatal resuscitation**	Airway suction apparatus: suction apparatus with catheter or suction bulb for mucus extraction. Observed available and reported functionality in service site.	x	x	x
	Neonatal resuscitation bag and mask (size 1 & size 0). Observed available and reported functionality in service site.	x	x	x
	Resuscitation table for newborn with heat source. Observed available and reported functional in service area.	x	x	x
	At least one staff in service area trained in newborn resuscitation using bag and mask in last 2 years.	x	x	x
	Facility provided neonatal resuscitation in past 12 months.	x	x	x
**Prevention of mother-to-child transmission of HIV (PMTCT)**	PMTCT room is a private room with auditory and visual privacy.**	x	x	x
	Antiretrovirals for newborns: nevirapine syrup or zidovudine syrup. Observed available and at least one unexpired in service site or where routinely stored.	x	x	x
	Antiretrovirals for mothers: country-specific first line ARV prophylaxis for HIV-positive pregnant women. Observed available and at least one unexpired in service site or where routinely stored.	x	x	x
	Cotrimoxazole syrup. Observed available and at least one unexpired in service site or where routinely stored.	x		x
	HIV diagnostic capacity: facility has the ability to conduct quantitative nucleic acid testing for HIV diagnosis onsite (PCR) or RNA / PCR for DNA-EID and has filter paper for DBS, or ELISA test with ELISA washer, ELISA reader, incubator, specific assay kit available and functional; Or facility sends specimen offsite for testing.	x	x	x
	PMTCT and infant and young child feeding (IYCF) guidelines available observed in service site. Calculated as proportion of guidelines available out of 2.	x		x
	At least one staff in service site trained in PMTCT in last two years.	x		x
	At least one staff in service site trained in newborn nutrition counseling of mother with HIV or IYCF in last 2 years.	x		x
**Kangaroo Mother Care (KMC)**	Bed/location for caregiver to stay overnight to provide KMC observed.	x	x	x
	Caps/hats for newborn observed available.	x	x	x
	Register to record KMC services observed available.	x	x	

	Guidelines, protocols or job aids for KMC observed available.	x	x	x
	At least one staff in service site trained in KMC in last two years.	x	x	x
	Facility provided KMC in last 3 months.	x	x	
**Detection and management of neonatal infection**	Antibiotic treatment for neonatal infection (Outpatient treatment: Gentamycin (IM or IV) and Amoxicillin (syrup/oral suspension) OR Inpatient treatment: (Benzylpenicillin (IV or IM) OR Ampicillin (IV or IM)) AND Gentamycin (IM or IV)) observed in pharmacy or anywhere in the facility where medicines are routinely stored; at least one with valid expiration date observed.	x	x	x
	Full blood count diagnostic capacity: Facility has the ability to conduct full blood count onsite and has functioning hematology analyzer or functioning blood chemistry analyzer available and functioning; or facility sends specimen offsite for testing.	x	x	x
	Chest x-ray capacity: Facility has the ability to conduct diagnostic x-ray testing + digital x-ray machine (not requiring film with equipment available) or x-ray machine and unexpired film and equipment available and functioning.	x		x
	Register for neonatal sepsis observed in service site.	x	x	x
	At least one staff in service site trained in neonatal sepsis in last two years.	x	x	x
	Guidelines/checklists for neonatal sepsis observed available in service site.	x	x	x
**Comfort and pain management**	Paracetamol syrup observed and at least one unexpired in service site or where routinely stored.	x		x
	Morphine (syrup or injectable) observed and at least one unexpired in service site or where routinely stored.	x		x
**Detection and management of hypoglycemia**	Glucose injectable solution (30% or 50%) observed and at least one unexpired in service site or where routinely stored.	x		x
	Blood glucose testing capacity: Facility has the ability to conduct blood glucose testing onsite and has glucometer and test strips available and functioning or blood chemistry analyzer available and functioning; or sends blood chemistry specimen offsite for testing.	x		x
**General facility readiness**	Thermometer: observed available and functional in service site.	x	x	x
	Infant weighing scale with 100g gradation. Observed available and reported functionality in service site.	x	x	x
	Stethoscope. Observed available and reported functionality in service site.	x	x	x

	Medication delivery mechanism: pediatric infusion kit + any IV fluid observed anywhere in the facility where supplies are routinely stored OR single use syringes observed in the delivery service provision area.	x	x	x
	Pulse oximeter. Observed available and reported functionality in service site.	x	x	x
	Oxygen and administration equipment: Oxygen currently in unit or oxygen currently not in unit, but report that unit calls centrally for oxygen if needed and humidifier, flowmeter, oxygen delivery apparatus are observed available and functioning in service site.	x	x	x
	Non-sharps waste container: Waste receptacle bin with lid and plastic bin liner for non-sharps infectious waste. Observed available in service site.	x	x	x
	Sharps container. Observed available in service site.	x	x	x
	Environmental disinfectant. Observed available in service site.	x	x	x
	Latex gloves: If equivalent non latex gloves are available this is acceptable; Either sterile or non sterile; latex or equivalent. Observed available in service site.	x	x	x
	Hand hygiene: soap and water, or alcohol based rub. Observed in service site.	x	x	x
	Guidelines for essential newborn care or referral of small or sick newborn observed available in service site.	x	x	x

*Based on country-specific recommendations: chlorhexidine for cord care excluded in Lusaka as it is not a national policy.
**In Nairobi, used outpatient room with auditory and visual privacy instead of PMTCT room, due to data unavailability.

Table S3. PCMC scale questions, response categories and scoring

Question	Response categories	PCMC scoring (out of 90)
**DIGNITY & RESPECT**		
Did the doctors, nurses, or other staff at the facility treat you with respect?	No, never	0
	Yes, a few times	1
	Yes, most of the time	2
	Yes, all the time	3
Did the doctors, nurses, or other staff at the facility treat you in a friendly manner?	No, never	0
	Yes, a few times	1
	Yes, most of the time	2
	Yes, all the time	3
Did you feel that they shouted at you, scolded, insulted, threatened, or talked to you rudely?	No, never	3
	Yes, once	2
	Yes, a few times	1
	Yes, many times	0
	Refused to respond	3
Did you feel like you were treated roughly like pushed, beaten, slapped, pinched, physically restrained, or gagged?	No, never	3
	Yes, once	2
	Yes, a few times	1
	Yes, many times	0
	Refused to respond	3
During examinations in the labor room, were you covered up with a cloth or blanket, or screened with a curtain so that you did not feel exposed?	No, never	0
	Yes, a few times	1
	Yes, most of the time	2
	Yes, all the time	3
Do you feel like your health information was or will be kept confidential at this facility?	No, never	0
	Yes, a few times	1
	Yes, most of the time	2

	Yes, all the time	3
**COMMUNICATION & AUTONOMY**		
During your time in the health facility did the doctors, nurses, or other health-care providers introduce themselves to you when they first came to see you?	No, none of them	0
	Yes, a few of them	1
	Yes, most of them	2
	Yes, all of them	3
Did the doctors, nurses, or other health-care providers call you by your name?	No, never	0
	Yes, a few times	1
	Yes, most of the time	2
	Yes, all the time	3
Did you feel like the doctors, nurses or other staff at the facility involved you in decisions about your care?	No, never	0
	Yes, a few times	1
	Yes, most of the time	2
	Yes, all the time	3
	Did not have to make any decisions	3
During the delivery, do you feel like you were able to be in the position of your choice?	No, never	0
	Yes, for a short time	1
	Yes, most of the time	2
	Yes, all the time	3
Did the doctors, nurses, or other staff at the facility speak to you in a language you could understand?	No, never	0
	Yes, a few times	1
	Yes, most of the time	2
	Yes, all the time	3
Did the doctors, nurses, or other staff at the facility ask your permission or consent before doing procedures on you?	No, never	0
	Yes, a few times	1
	Yes, most of the time	2
	Yes, all the time	3
Did the doctors, nurses, or other staff at the facility explain to you why they were doing examinations or procedures on you?	No, never	0
	Yes, a few times	1
	Yes, most of the time	2

	Yes, all the time	3
Did the doctors, nurses, or other staff at the facility explain to you why they were giving you any medicine?	No, never	0
	Yes, a few times	1
	Yes, most of the time	2
	Yes, all the time	3
	Did not get any medicine	3
Did you feel you could ask the doctors, nurses, or other staff at the facility any questions you had?	No, never	0
	Yes, a few times	1
	Yes, most of the time	2
	Yes, all the time	3
**SUPPORTIVE CARE**		
How did you feel about the amount of time you waited to receive care? Would you say it was:	Very short	3
	Somewhat short	2
	Somewhat long	1
	Very long	0
Did the doctors and nurses at the facility show concern for your feelings about your delivery?	No, never	0
	Yes, a few times	1
	Yes, most of the time	2
	Yes, all the time	3
Did the doctors, nurses, or other staff at the facility try to understand your anxieties?	No, never	0
	Yes, a few times	1
	Yes, most of the time	2
	Yes, all the time	3
	Did not have any anxiety	3
When you needed help, did you feel the doctors, nurses, or other staff at the facility paid attention?	No, never	0
	Yes, a few times	1
	Yes, most of the time	2
	Yes, all the time	3
Do you feel the doctors or nurses did everything they could to help control your pain?	No, never	0
	Yes, a few times	1
	Yes, most of the time	2

	Yes, all the time	3
Were you allowed to have someone you wanted (outside of staff at the facility, such as family or friends) to stay with you during labor?	No, never	0
	Yes, a few times	1
	Yes, most of the time	2
	Yes, all the time	3
	I did not want someone to stay with me	3
Were you allowed to have someone you wanted to stay with you during delivery?	No, never	0
	Yes, a few times	1
	Yes, most of the time	2
	Yes, all the time	3
	I did not want someone to stay with me	3
Did you feel the doctors, nurses, or other staff at the facility took good care of you, at the best of their ability?	No, never	0
	Yes, a few times	1
	Yes, most of the time	2
	Yes, all the time	3
Did you feel you could completely trust the doctors, nurses, or other staff at the facility with regards to your care?	No, never	0
	Yes, a few times	1
	Yes, most of the time	2
	Yes, all the time	3
Do you think there were enough health staff in the facility to care for you?	No, never	0
	Yes, a few times	1
	Yes, most of the time	2
	Yes, all the time	3
Thinking about the labor and postnatal wards, did you feel the health facility was crowded?	No, never	3
	Yes, a few times	2
	Yes, most of the time	1
	Yes, all the time	0
Thinking about the wards, washrooms, and the general environment of the health facility, would you	Very dirty	0
	Dirty	1

say the facility was very clean, clean, dirty, or very dirty?	Clean	2
	Very clean	3
Was there running water in the facility?	No, never	0
	Yes, a few times	1
	Yes, most of the time	2
	Yes, all the time	3
Was there electricity in the facility?	No, never	0
	Yes, a few times	1
	Yes, most of the time	2
	Yes, all the time	3
In general, did you feel safe in the health facility?	No, never	0
	Yes, a few times	1
	Yes, most of the time	2
	Yes, all the time	3

Table S4. Service availability in facilities offering maternal and newborn health services by city.

	LUSAKA				NAIROBI		OUAGADOUGOU			
	Serving any informal settlement		Not serving informal settlements		Serving informal settlements		Serving informal settlements		Not serving informal settlements	
	(%)	n	(%)	n	(%)	n	(%)	n	(%)	n
**Labor & Delivery Care**	35.5	31	30.7	88	47.4	38	47.0	115	39.1	215
BEmONC (facility reported)	32.3	31	29.6	88	47.4	38	41.7	115	36.3	215
Parenteral administration of antibiotics (IV or IM) for mothers	32.3	31	29.6	88	47.4	38	44.3	115	38.1	215
Parenteral administration of anticonvulsants (magnesium sulfate) for management of pre-eclampsia and eclampsia (IV or IM)	32.3	31	29.6	88	42.1	38	18.3	115	19.1	215
Parenteral administration of oxytocic for treatment of postpartum haemorrhage (IV or IM)	32.3	31	30.7	88	42.1	38	44.3	115	37.7	215
Removal of retained products of conception using D&C or manual vacuum aspiration	16.1	31	25.0	88	31.6	38	33.9	115	24.7	215
Assisted vaginal delivery using manual vacuum extraction (MVE) or forceps	12.9	31	20.5	88	23.7	38	29.6	115	23.7	215
Manual removal of placenta	32.3	31	30.7	88	47.4	38	44.3	115	35.8	215
Neonatal resuscitation with bag and mask	32.3	31	30.7	88	44.7	38	21.7	115	17.7	215
**All 7 BEmONC signal functions**	9.7	31	20.5	88	15.8	38	9.6	115	4.7	215
Oxygen administration	32.3	31	30.7	88	39.5	38	5.2	115	9.3	215
CEmONC (among hospitals, facility reported)	100.0	4	77.8	9	66.7	3	23.5	17	27.8	72
Blood transfusion (among hospitals)	75.0	4	55.6	9	66.7	3	23.5	17	22.2	72
C section (among hospitals)	75.0	4	55.6	9	66.7	3	23.5	17	25.0	72
**Maternal And Well Infant Postpartum/ Postnatal Care (inpatient, outpatient or both)***	71.0	31	62.5	88	68.4	38	49.6	115	42.3	215
Counseling on family planning	64.5	31	58.0	88	65.8	38	47.0	115	42.3	215
Provision of newborn vaccines (BCG & OPV0)	54.8	31	45.5	88	55.3	38	37.4	115	29.8	215

Counselling on child nutritional needs and good feeding practices	64.5	31	55.7	88	63.2	38	48.7	115	41.4	215
Counselling on danger signs in the newborn	64.5	31	56.8	88	65.8	38	49.6	115	42.3	215
Counselling on cord care and hygiene	61.3	31	56.8	88	63.2	38	49.6	115	42.3	215
Counselling on child immunization needs	64.5	31	53.4	88	63.2	38	48.7	115	40.9	215
**Small or Sick Newborn Care**	54.8	31	43.2	88	86.8	38	13.0	115	20.0	215
Kangaroo Mother Care	16.1	31	18.2	88	10.5	38	8.7	115	12.6	215
Services or referral for cases of neonatal sepsis	38.7	31	29.6	88	73.7	38	12.2	115	17.2	215
Neonatal resuscitation	32.3	31	30.7	88	44.7	38	21.7	115	17.7	215
Prevention of mother-to-child transmission of HIV	35.5	31	27.3	88	42.1	38	37.4	115	31.6	215
Early initiation and support for breastfeeding	35.5	31	30.7	88	47.4	38	47.0	115	38.6	215

**routinely provided to all clients*

Figure S1: Distribution of labor & delivery readiness scores comparing facilities serving and not serving informal settlements, by city.

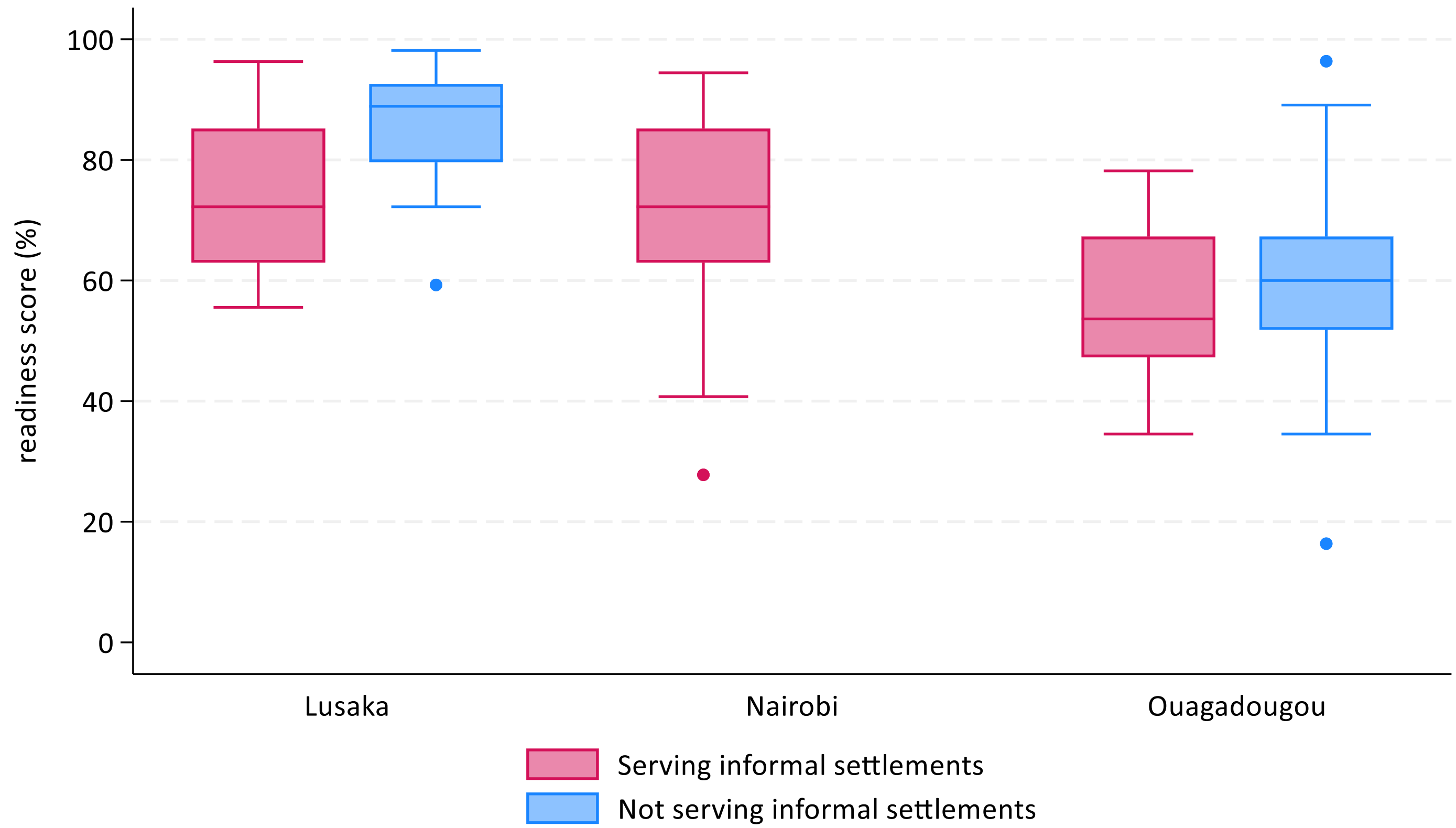

Table S5. Facility readiness scores (%) among facilities offering labor and delivery care by city.

	LUSAKA			NAIROBI	OUAGADOUGOU		
Domain	Serving informal settlements (n=11) % (SD)	Not serving informal settlements (n=27) % (SD)	t test p value	Serving informal settlements (n=18) % (SD)	Serving informal settlements (n=54) % (SD)	Not serving informal settlements (n=84) % (SD)	t test p value
Labor & delivery care							
Overall labor & delivery readiness *(simple additive score)*	73.6 (12.5)	85.6 (9.2)	**0.002**	69.5 (19.4)	55.9 (11.9)	60.3 (12.7)	**0.044**
Equipment	77.9 (12.7)	93.2 (9.4)	**<0.001**	65.7 (23.8)	61.1 (13.6)	65.7 (13.0)	0.050
Medicines & commodities	61.5 (17.9)	84.3 (12.9)	**<0.001**	72.2 (23.2)	46.7 (17.9)	52.0 (22.1)	0.145
Basic amenities	79.5 (18.8)	90.7 (12.3)	**0.036**	77.8 (19.0)	70.8 (18.7)	72.0 (17.5)	0.705
Human resources & guidelines	75.3 (26.4)	57.1 (24.7)	0.051	68.3 (15.1)	32.5 (25.0)	40.8 (26.4)	0.069
Performance of BEmONC signal function in last 12 months	76.6 (19.5)	88.4% (20.2)	0.110	73.8 (21.5)	72.0 (23.2)	71.9 (19.0)	0.996
% facilities with score ≥95% (ceiling)	*9.1*	*7.4*		*0.0*	*0.0*	*1.2*	
% facilities with score ≤5% (floor)	*0.0*	*0.0*		*0.0*	*0.0*	*0.0*	
Small & sick newborn care							
Overall SSNC readiness *(weighted additive score)*	60.8 (14.5)	70.7 (11.4)	**0.032**	61.3 (17.9)	37.2 (9.3)	40.3 (12.4)	0.100
Immediate newborn care and routine care	54.8 (18.4)	82.2 (20.0)	**<0.001**	66.3 (25.7)	52.0 (18.2)	55.0 (18.8)	0.366
Early initiation and support for breastfeeding	81.8 (27.3)	60.5 (29.3)	**0.045**	74.1 (21.6)	55.6 (25.9)	61.5 (27.6)	0.208

Neonatal resuscitation	80.0 (31.0)	90.4 (12.9)	0.305	77.8 (25.6)	35.2 (22.0)	40.5 (24.0)	0.194
Prevention of mother-to-child transmission of HIV	85.8 (19.0)	70.6 (21.4)	**0.047**	77.8 (28.3)	41.8 (18.7)	43.5 (19.6)	0.620
Kangaroo Mother Care	33.3 (34.2)	27.2 (32.1)	0.601	14.8 (29.1)	1.9 (6.6)	3.9 (14.3)	0.264
Detection and management of neonatal infection	43.9 (25.0)	51.9 (21.4)	0.331	42.2 (23.7)	20.7 (11.6)	24.8 (19.6)	0.124
Comfort and pain management	13.6 (23.4)	66.7 (34.0)	**<0.001**	NA	38.9 (21.0)	35.7 (26.4)	0.458
Detection and management of hypoglycemia	63.6 (39.3)	94.4 (16.0)	**0.028**	NA	16.7 (27.5)	20.2 (31.2)	0.493
General facility readiness	90.2 (9.7)	92.3 (8.0)	0.488	75.9 (20.8)	72.5 (10.4)	77.8 (10.0)	**0.004**
% facilities with score ≥95% (ceiling)	*0.0*	*0.0*		*0.0*	*0.0*	*0.0*	
% facilities with score ≤5% (floor)	*0.0*	*0.0*		*0.0*	*0.0*	*0.0*	

Figure S2 Panels A-C. Radar plots of labor & delivery care item availability by city.

A. Lusaka: facilities serving informal settlements (n=11)

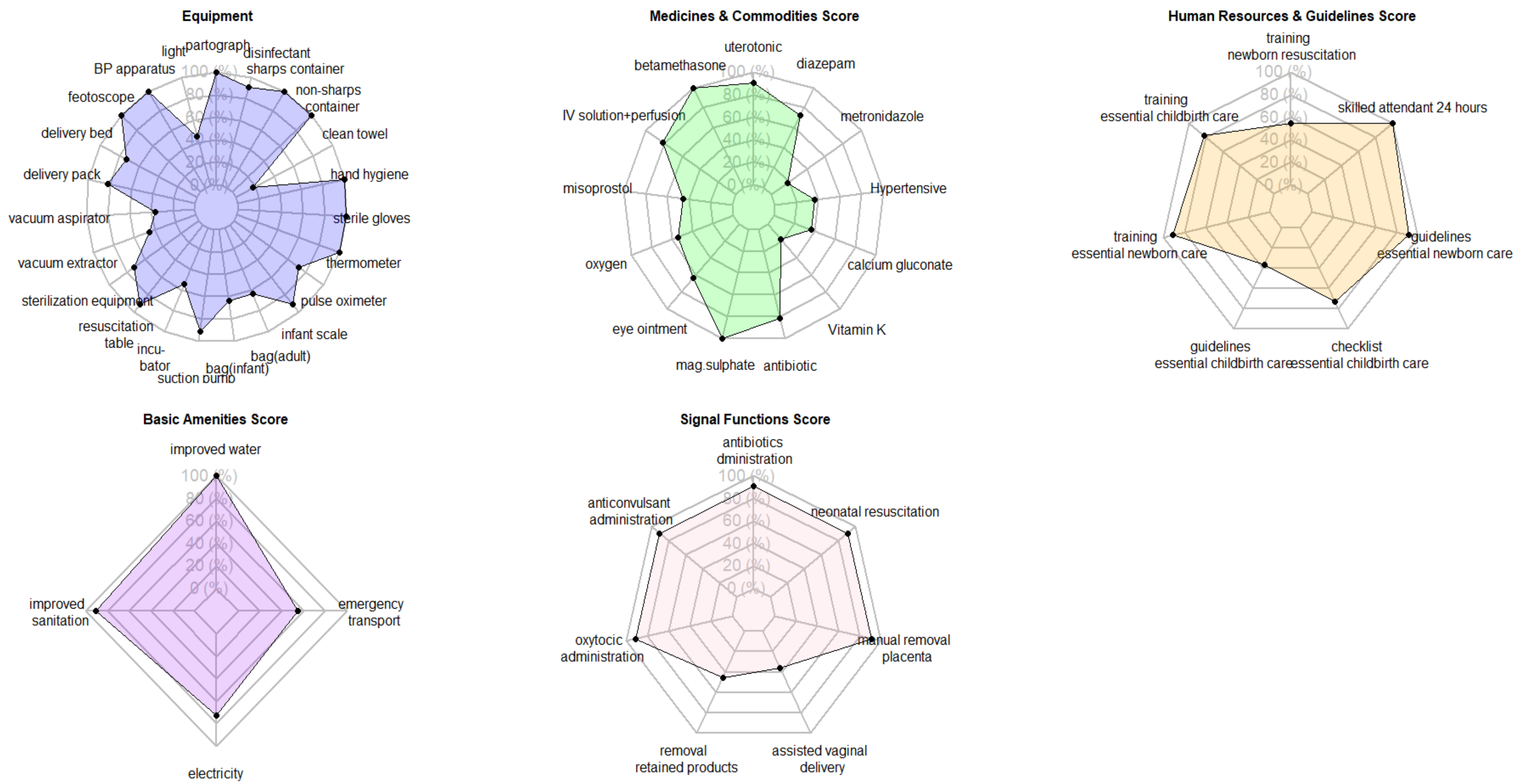

B. Nairobi: facilities serving informal settlements (n=18)

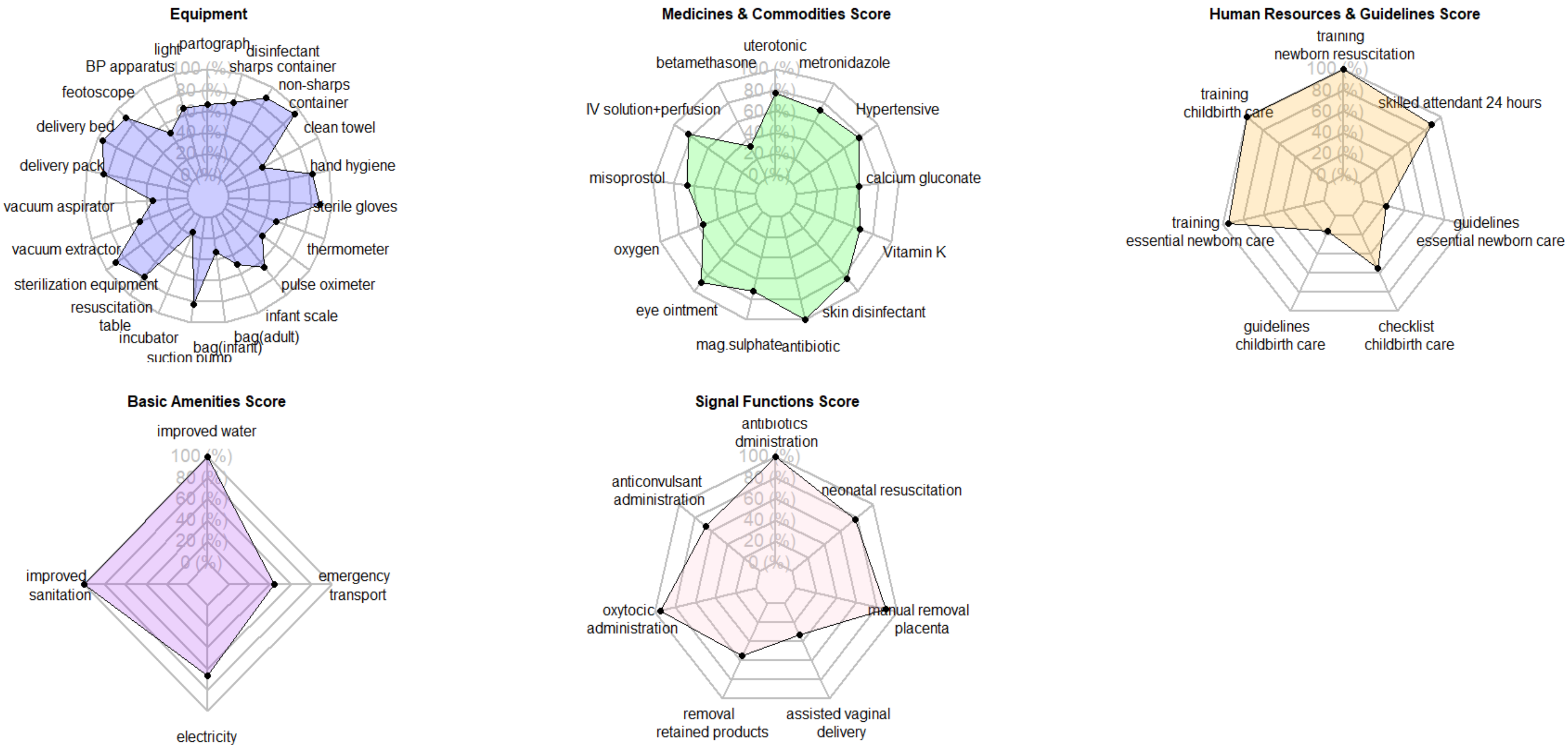

C. Ouagadougou: facilities serving informal settlements (n=54)

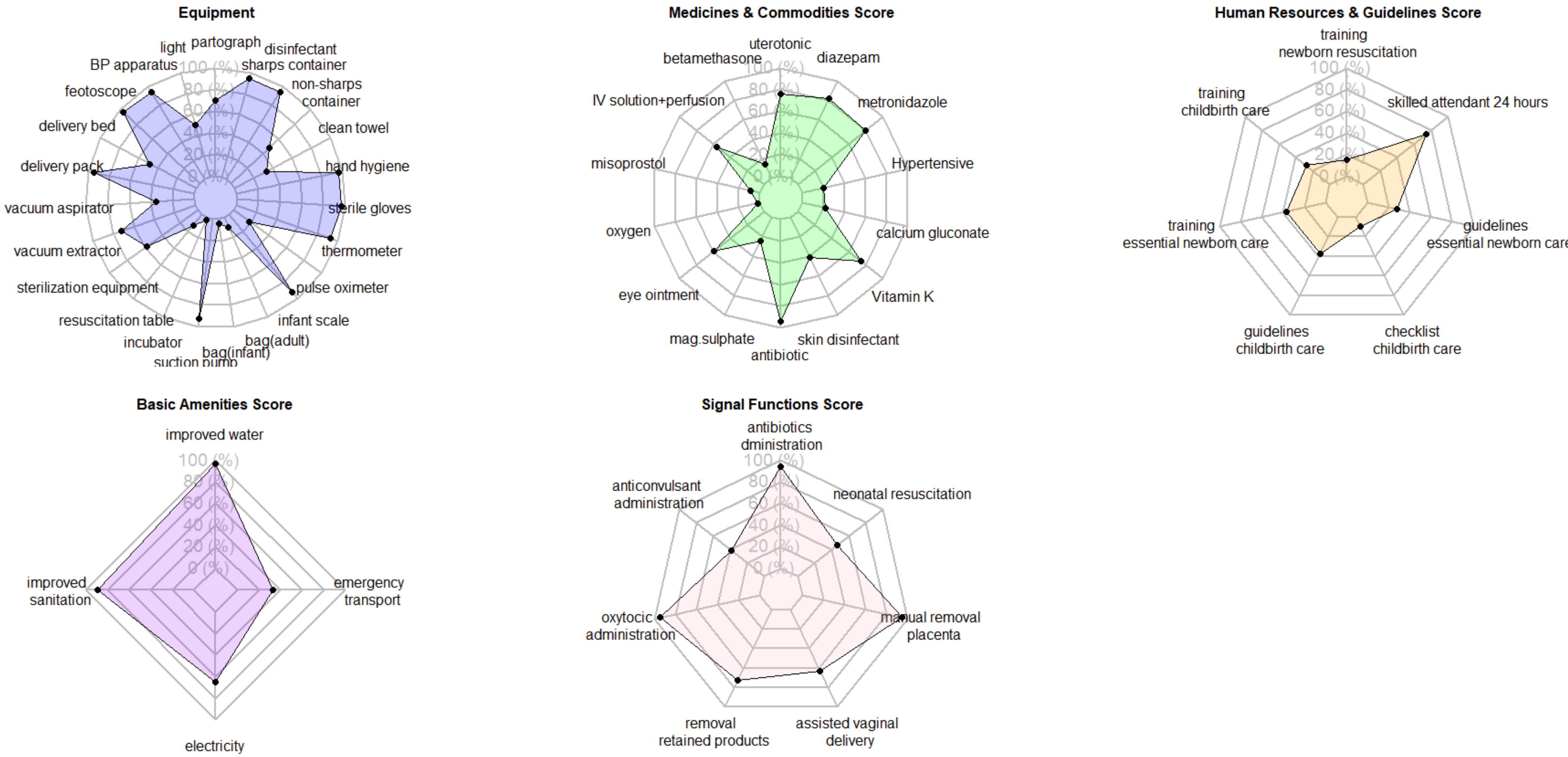

Table S6. Item availability for labor & delivery care readiness by city and facilities serving/not serving informal settlements.

		LUSAKA		NAIROBI	OUAGADOUGOU	
Domain	Item	Serving informal settlements (n=11)	Not serving informal settlements (n=27)	Serving informal settlements (n=18)	Serving informal settlements (n=54)	Not serving informal settlements (n=84)
Equipment	Blank partograph	100.0%	96.3%	66.7%	70.4%	71.4%
	Examination light	45.5%	92.6%	66.7%	50.0%	61.9%
	Blood pressure apparatus	100.0%	100.0%	50.0%	94.4%	95.2%
	Foetal stethoscope	100.0%	100.0%	88.9%	96.3%	97.6%
	Delivery bed	72.7%	96.3%	94.4%	48.1%	60.7%
	Delivery pack (or cord clamp, episiotomy scissors, scissors/blade to cut cord, suture material with needle, and needle holder)	81.8%	100.0%	83.3%	94.4%	90.5%
	Vacuum aspirator or D&C kit and speculum	36.4%	74.1%	33.3%	35.2%	36.9%
	Manual vacuum extractor or forceps	45.5%	85.2%	50.0%	72.2%	81.0%
	Sterilization equipment	72.7%	77.8%	88.9%	57.4%	69.0%
	Resuscitation table with heat source	90.9%	100.0%	77.8%	13.0%	22.6%
	Infant incubator	54.5%	81.5%	16.7%	1.9%	9.5%
	Suction pump and catheter or suction bulb (newborn)	90.9%	96.3%	83.3%	92.6%	91.7%
	Neonatal resuscitation bag and mask (size 1 & size 0)	63.6%	88.9%	33.3%	3.7%	7.1%
	Adult resuscitation bag and mask	63.6%	85.2%	50.0%	9.3%	14.3%
	Infant weighing scale (100g gradation)	90.9%	96.3%	66.7%	92.6%	92.9%
	Pulse oximeter	72.7%	92.6%	44.4%	18.5%	27.4%
	Thermometer	100.0%	100.0%	50.0%	92.6%	95.2%
	Sterile gloves	100.0%	100.0%	88.9%	96.3%	98.8%
	Hand hygiene	100.0%	100.0%	83.3%	96.3%	94.0%
	Clean towel for drying newborn	18.2%	85.2%	38.9%	33.3%	32.1%

	Non-sharps waste container	100.0%	96.3%	94.4%	48.1%	67.9%
	Sharps container	100.0%	100.0%	88.9%	94.4%	96.4%
	Environmental disinfectant	90.9%	100.0%	72.2%	94.4%	96.4%
Medicines & commodities	Injectable uterotonic	90.9%	70.4%	77.8%	75.9%	73.8%
	Injectable beta/dexamethasone	100.0%	100.0%	33.3%	14.8%	22.6%
	IV solution and perfusion kit	81.8%	70.4%	83.3%	55.6%	67.9%
	Misoprostol	45.5%	85.2%	66.7%	9.3%	28.6%
	Oxygen and administration equipment	54.5%	70.4%	55.6%	1.9%	9.5%
	Antibiotic eye ointment (tetracycline)	63.6%	81.5%	88.9%	59.3%	66.7%
	Injectable magnesium sulphate	100.0%	88.9%	72.2%	24.1%	39.3%
	Injectable antibiotic for maternal/neonatal sepsis	81.8%	100.0%	100.0%	94.4%	89.3%
	Skin disinfectant for cord care (chlorhexidine gel)	N/A	N/A	83.3%	40.7%	52.4%
	Injectable Vitamin K	18.2%	77.8%	66.7%	74.1%	67.9%
	Injectable calcium gluconate	36.4%	81.5%	61.1%	22.2%	23.8%
	Antihypertensive (hydralazine, nifedipine, or methyldopa)	36.4%	85.2%	77.8%	20.4%	33.3%
	Injectable metronidazole	18.2%	92.6%	72.2%	79.6%	73.8%
	Injectable diazepam	72.7%	92.6%	N/A	81.5%	78.6%
Human resources and guidelines	Staff trained in newborn resuscitation	54.5%	59.3%	100.0%	14.8%	27.4%
	Staff trained in essential childbirth care	81.8%	63.0%	100.0%	27.8%	36.9%
	Staff trained in essential newborn care	90.9%	66.7%	94.4%	37.0%	52.4%
	Guidelines for essential childbirth care	36.4%	40.7%	16.7%	37.0%	35.7%
	Checklist/job aids for essential childbirth care	72.7%	37.0%	55.6%	9.3%	8.3%
	Guidelines for essential newborn care	90.9%	44.4%	22.2%	27.8%	39.3%
	Skilled attendant present on call (onsite or in near proximity) 24 hours	100.0%	88.9%	88.9%	74.1%	85.7%
Basic amenities	Improved water source	100.0%	100.0%	100.0%	96.3%	97.6%
	Improved sanitation facility	90.9%	100.0%	100.0%	88.9%	94.0%
	Electricity available at all times when facility was open in last 7 days and/or back-up energy	72.7%	96.3%	66.7%	64.8%	61.9%
	Emergency transport (available on day of survey)	54.5%	66.7%	44.4%	33.3%	34.5%

Performance of BEmONC signal functions in last 12 months	Parenteral administration of antibiotics (IV or IM) for mothers	90.9%	92.6%	100.0%	94.4%	97.6%
	Parenteral administration of anticonvulsants (magnesium sulfate) for management of pre-eclampsia and eclampsia (IV or IM)	90.9%	92.6%	66.7%	38.9%	48.8%
	Parenteral administration of oxytocic for treatment of postpartum haemorrhage (IV or IM)	90.9%	96.3%	94.4%	94.4%	96.4%
	Removal of retained products of conception using D&C or manual vacuum aspiration	45.5%	77.8%	55.6%	72.2%	63.1%
	Assisted vaginal delivery using manual vacuum extraction (MVE) or forceps	36.4%	63.0%	33.3%	63.0%	60.7%
	Manual removal of placenta	90.9%	96.3%	88.9%	94.4%	91.7%
	Neonatal resuscitation with bag and mask	90.9%	100.0%	77.8%	46.3%	45.2%

Figure S3. Labor & delivery care readiness scores (%) by facility type and managing authority

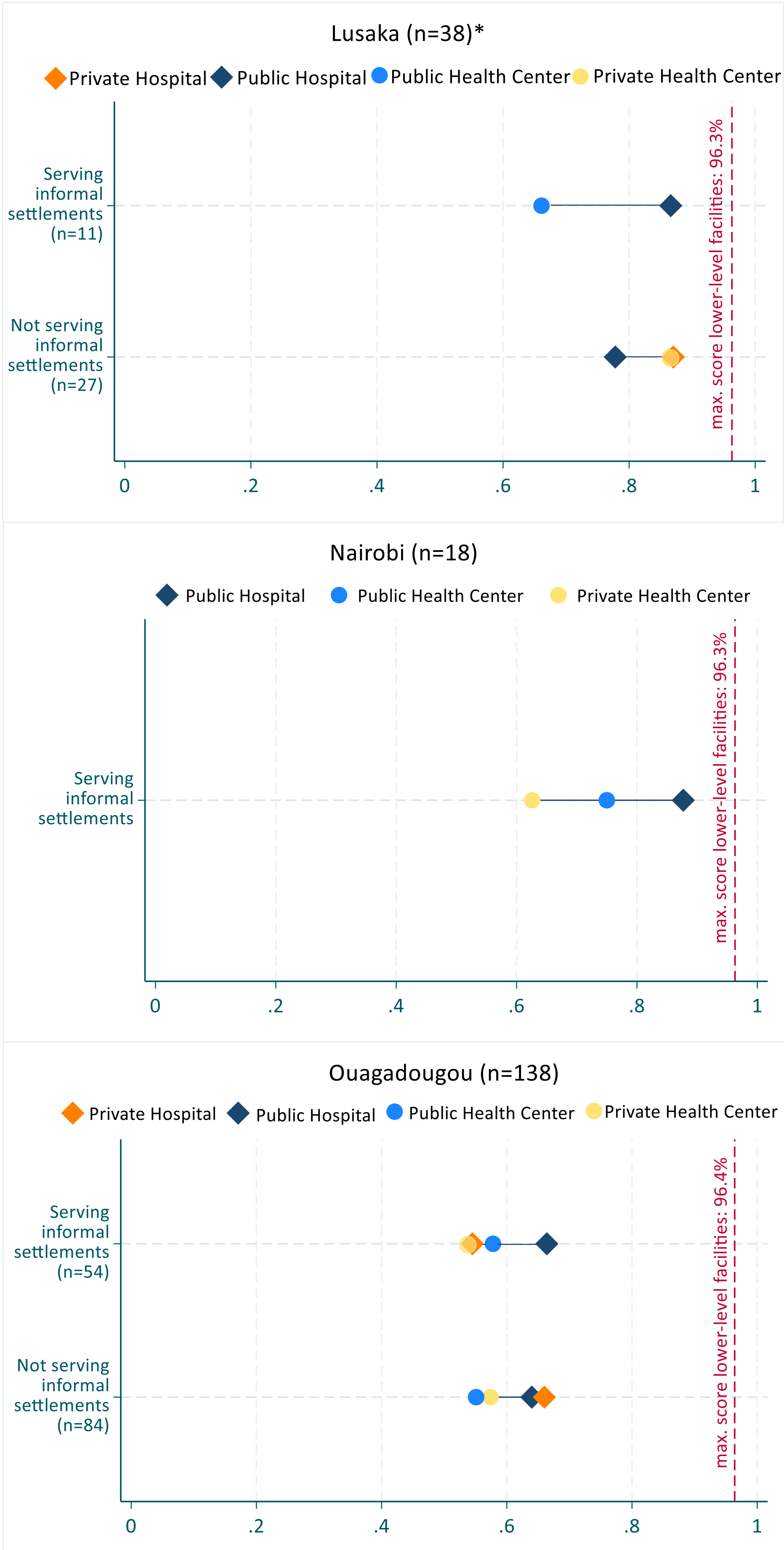

**in Lusaka there are no private facilities serving informal settlements, and there were no public health centers in areas not serving informal settlements.*

Note: The red dashed line represents the maximum readiness score for lower-level facilities, based on exclusion of infant incubator and resuscitation table, and was calculated as 96.3% in Nairobi and Lusaka, and 96.4% in Ouagadougou.

Table S7. Facility readiness scores by facility type and managing authority.

		LUSAKA		NAIROBI	OUAGADOUGOU	
		Serving informal settlements (n=11) % SD	Not serving informal settlements (n=27) % SD	Serving informal settlements (n=18) % SD	Serving informal settlements (n=54) % SD	Not serving informal settlements (n=84) % SD
**Labor & Delivery Care**	Public Health Center	66.1% SD=6.8 *(n=7)*	*N/A*	75.0% SD=9.8 *(n=4)*	57.8% SD=11.9 *(n=18)*	55.1% SD=11.3 *(n=28)*
	Private Health Center	*N/A*	86.5% SD=9.1 *(n=19)*	62.6% SD=21.0 *(n=11)*	54.2% SD=12.4 *(n=24)*	57.4% SD=10.8 *(n=18)*
	Public Hospital	86.6% SD=8.4 *(n=4)*	77.8% SD=9.6 *(n=3)*	87.7% SD=3.8 *(n=3)*	66.4% SD=6.4 *(n=2)*	64.0% SD=13.0 *(n=10)*
	Private Hospital	*N/A*	87.0% SD=9.0 *(n=5)*	*N/A*	54.5% SD=11.5 *(n=10)*	66.0% SD=12.9 *(n=28)*
	*One-way ANOVA p- value**	***p=0.002***	*p=0.305*	*p=0.109*	*p=0.466*	***p=0.005***
**Small & Sick Newborn Care**	Public Health Center	52.5% SD=11.1 (n=7)	*N/A*	64.2% SD=10.5 *(n=4)*	39.1% SD=7.3 *(n=18)*	37.5% SD=10.2 *(n=28)*
	Private Health Center	*N/A*	71.6% SD=11.0 *(n=19)*	56.0% SD=20.0 *(n=11)*	34.5% SD=8.6 *(n=24)*	35.1% SD=9.2 *(n=18)*
	Public Hospital	75.3% SD=4.0 (n=4)	62.2% SD=9.0 *(n=3)*	76.7% SD=5.5 *(n=3)*	49.3% SD=8.7 *(n=2)*	44.6% SD=12.2 *(n=10)*
	Private Hospital	*N/A*	72.1% SD=14.2 *(n=5)*	*N/A*	38.1% SD=12.0 *(n=10)*	44.9% SD=14.6 *(n=28)*
	*One-way ANOVA p- value**	***p=0.004***	*p=0.411*	*p=0.202*	*p=0.095*	***p=0.019***

**one-way analysis of variance (ANOVA) p<0.05 suggests that at least one of the mean scores is statistically significantly different, across facility types and managing authority.*

Figure S4. Distribution of small and sick newborn readiness scores comparing facilities serving and not serving informal settlements, by city

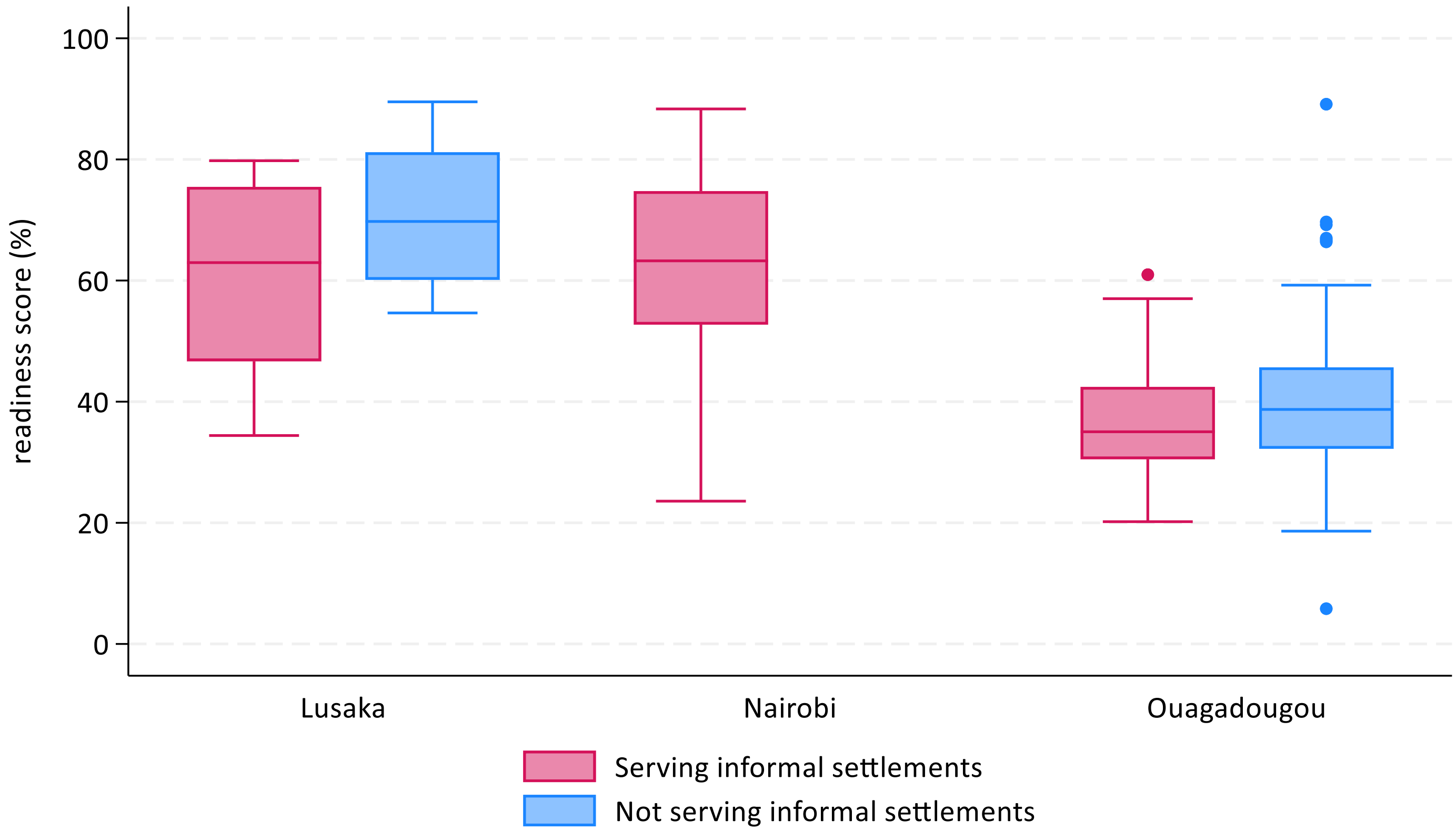

Figure S5 Panels A-C: Radar plots of small & sick newborn care item availability by city.

A. Lusaka: facilities serving informal settlements (n=11)

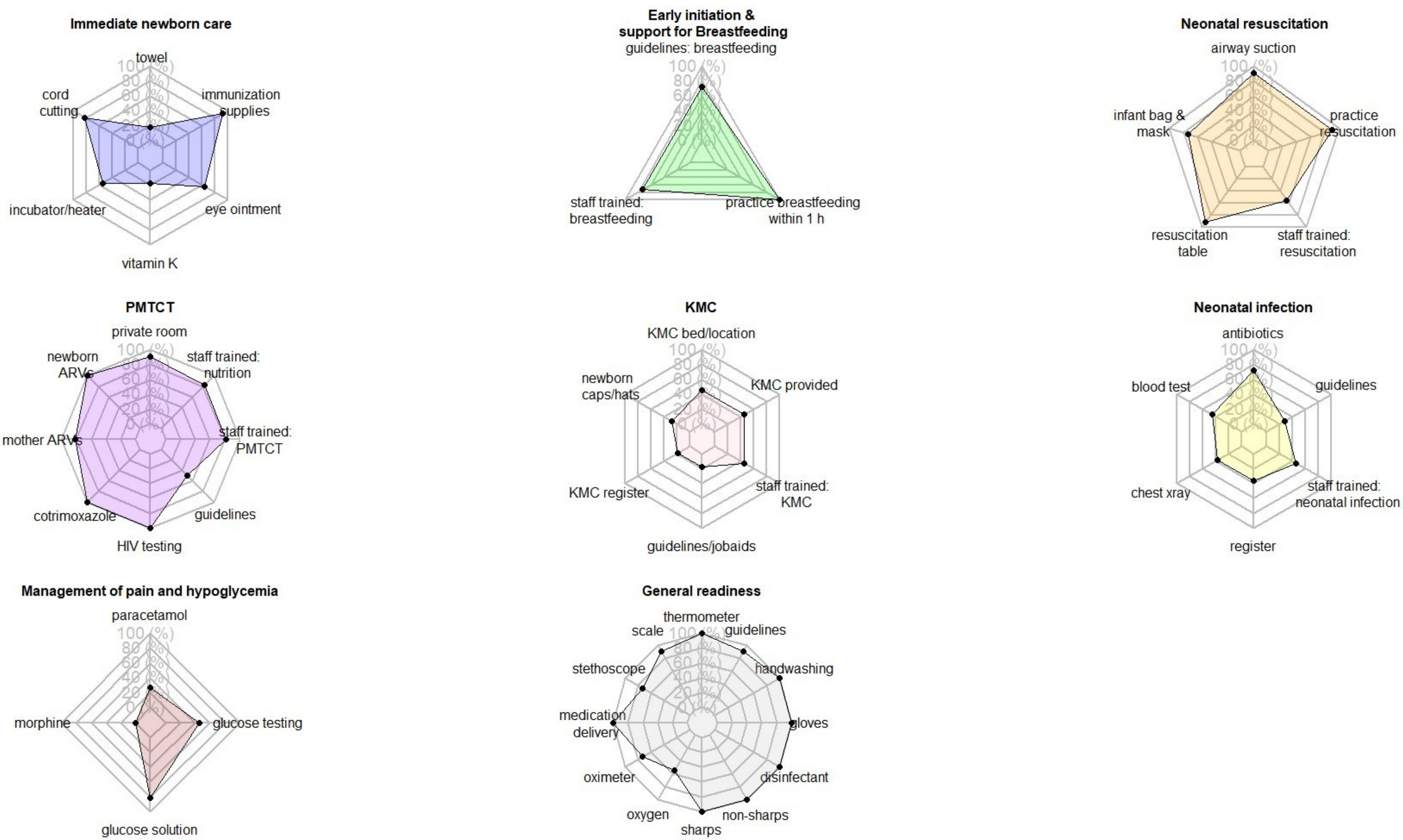

B. Nairobi: facilities serving study area (n=18)

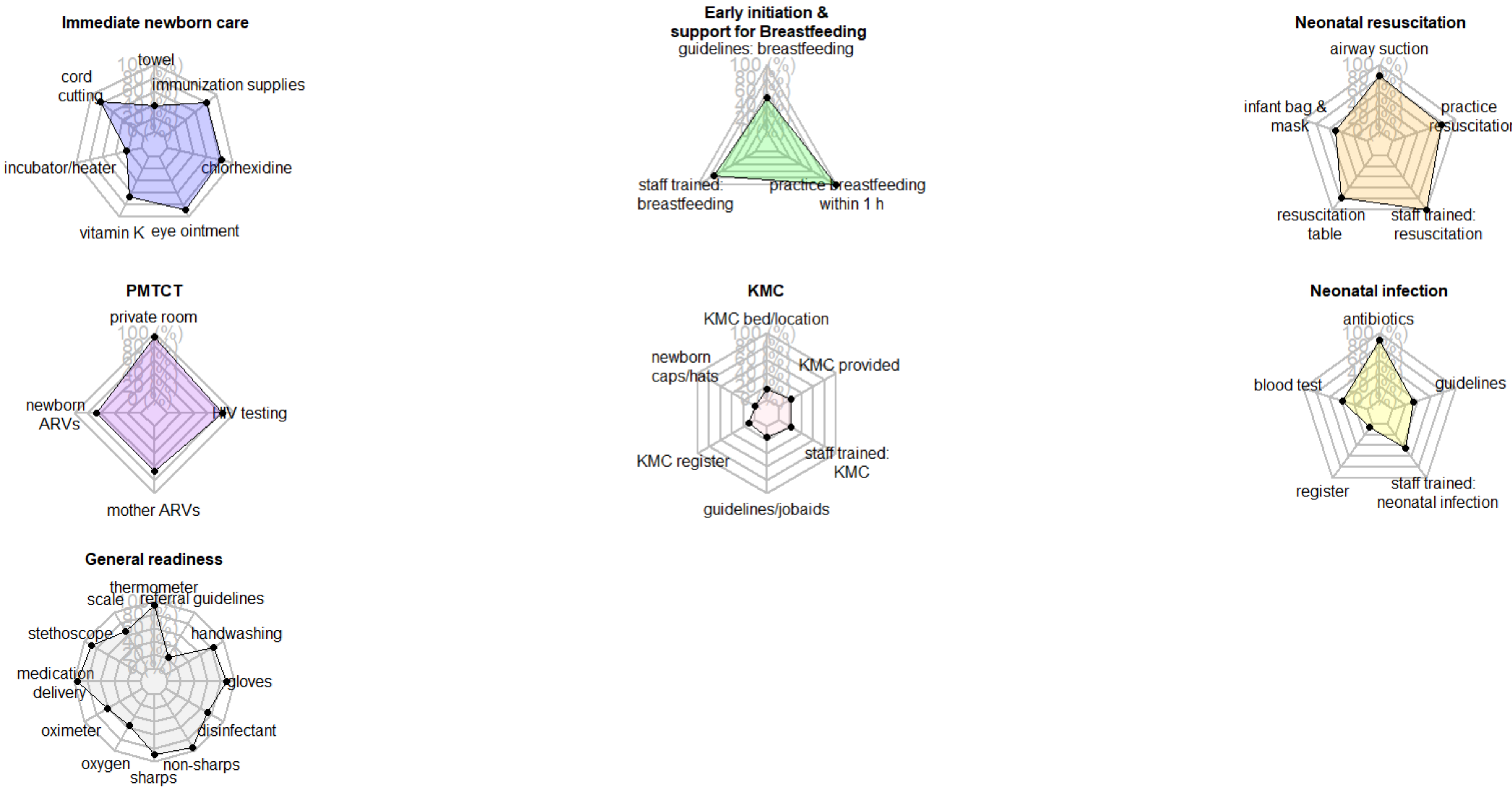

C. Ouagadougou: facilities serving informal settlements (n=54)

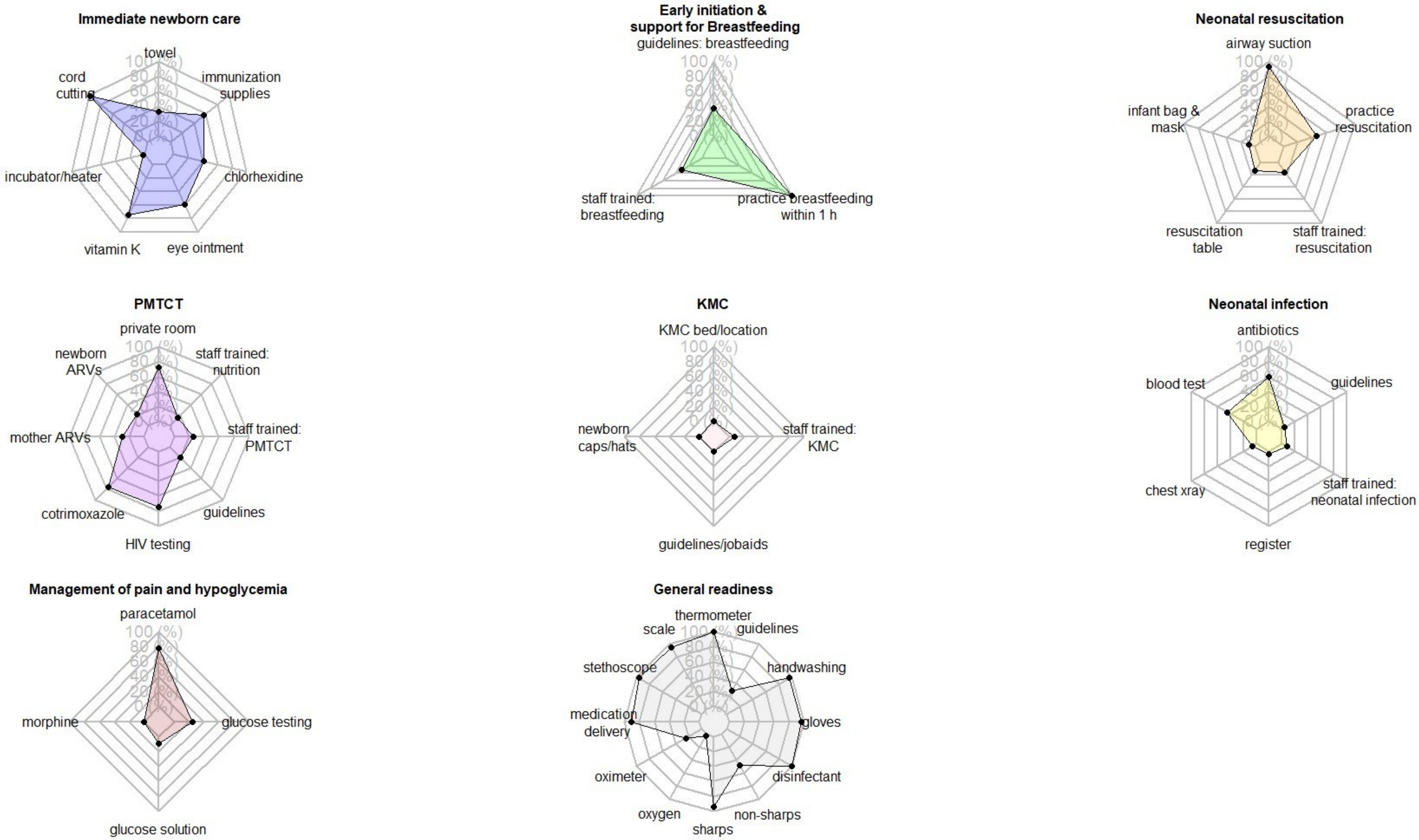

Table S8. Item availability for small & sick newborn care readiness by city and facilities serving/not serving informal settlements.

		LUSAKA		NAIROBI	OUAGADOUGOU	
Domain	Item	Serving informal settlements (n=11)	Not serving informal settlements (n=27)	Serving informal settlements (n=18)	Serving informal settlements (n=54)	Not serving informal settlements (n=84)
Immediate newborn care and routine care	Clean towel for drying newborn,	18.2%	85.2%	38.9%	33.3%	32.1%
	Cord cutting supplies	81.8%	100.0%	83.3%	98.1%	92.9%
	Radiant heater/warmth source or infant incubator	54.5%	81.5%	22.2%	1.9%	11.9%
	Vitamin K (inj)	18.2%	77.8%	66.7%	74.1%	67.9%
	Antibiotic eye ointment	63.6%	81.5%	88.9%	59.3%	66.7%
	Chlorhexidine solution	N/A	N/A	83.3%	40.7%	52.4%
	Immunization supplies	92.4%	67.3%	80.6%	56.8%	60.9%
Early initiation and support for breastfeeding	Guidelines for breastfeeding and promotion of breastfeeding	72.7%	40.7%	50.0%	37.0%	45.2%
	Staff trained in breastfeeding and promotion of breastfeeding	72.7%	40.7%	72.2%	29.6%	40.5%
	Routine practice of breastfeeding initiation within 1 hour of birth	100.0%	100.0%	100.0%	100.0%	98.8%
Neonatal resuscitation	Airway suction apparatus	90.9%	96.3%	83.3%	92.6%	92.9%
	Infant resuscitation bag & mask	72.7%	96.3%	50.0%	9.3%	11.9%
	Resuscitation table with heat source	90.9%	100.0%	77.8%	13.0%	25.0%
	Staff trained in neonatal resuscitation using bag and mask	54.5%	59.3%	100.0%	14.8%	27.4%
	Facility provided neonatal resuscitation in past three months	90.9%	100.0%	77.8%	46.3%	45.2%
Prevention of mother-to-child transmission of HIV (PMTCT)	PMTCT room is private room with auditory and visual privacy	90.9%	88.9%	94.4%	72.2%	73.8%
	Antiretrovirals for newborns	100.0%	81.5%	66.7%	22.2%	31.0%

	Antiretrovirals for mothers	81.8%	37.0%	66.7%	29.6%	33.3%
	Cotrimoxazole	100.0%	96.3%	N/A	75.9%	71.4%
	HIV diagnostic capacity	100.0%	92.6%	83.3%	74.1%	79.8%
	PMTCT and infant and young child feeding (IYCF) guidelines	50.0%	42.6%	N/A	19.4%	27.4%
	Staff trained in PMTCT in last 2 years	81.8%	55.6%	N/A	25.9%	17.9%
	Staff trained in newborn nutrition counseling of mother with HIV or IYCF in last years.	81.8%	70.4%	N/A	14.8%	13.1%
Kangaroo Mother Care (KMC)	Bed/location for caregiver to stay overnight to provide KMC	45.5%	37.0%	16.7%	0.0%	2.4%
	Caps/hats for newborn	27.3%	33.3%	0.0%	0.0%	3.6%
	Register to record KMC services	18.2%	7.4%	11.1%	N/A	N/A
	Guidelines, protocols or job aids for KMC,	18.2%	29.6%	16.7%	0.0%	3.6%
	Staff trained in KMC in last 2 years,	45.5%	33.3%	22.2%	7.4%	6.0%
	Facility provided KMC in last 3 months.	45.5%	22.2%	22.2%	N/A	N/A
Detection and management of neonatal infection	First line antibiotics for neonatal sepsis OR second line antibiotics for neonatal sepsis,	72.7%	100.0%	88.9%	59.3%	69.0%
	Full blood count diagnostic capacity,	45.5%	66.7%	38.9%	44.4%	45.2%
	Chest x-ray capacity	36.4%	63.0%	N/A	5.6%	2.4%
	Register for neonatal sepsis	36.4%	14.8%	5.6%	3.7%	9.5%
	Staff trained in neonatal sepsis in last 2 years	45.5%	33.3%	44.4%	7.4%	13.1%
	Guidelines/checklists for neonatal sepsis	27.3%	33.3%	33.3%	3.7%	9.5%
Comfort and pain management	Paracetamol syrup	27.3%	88.9%	N/A	77.8%	65.5%
	Morphine (syrup or injectable)	0.0%	44.4%	N/A	0.0%	6.0%
Detection and management of hypoglycemia	Glucose injectable solution,	81.8%	100.0%	N/A	9.3%	7.1%
	Blood glucose testing capacity	45.5%	88.9%	N/A	24.1%	33.3%
General facility readiness	Thermometer	100.0%	100.0%	94.4%	100.0%	100.0%
	Infant scale	90.9%	100.0%	66.7%	94.4%	97.6%
	Stethoscope	72.7%	96.3%	88.9%	96.3%	97.6%
	Medication delivery mechanism	100.0%	100.0%	94.4%	90.7%	94.0%
	Pulse oximeter	72.7%	100.0%	61.1%	24.1%	39.3%
	Oxygen and administration equipment	54.5%	70.4%	55.6%	1.9%	9.5%

	Non-sharps waste container	100.0%	96.3%	94.4%	48.1%	67.9%
	Sharps container	100.0%	100.0%	88.9%	94.4%	96.4%
	Environmental disinfectant	100.0%	100.0%	72.2%	100.0%	98.8%
	Sterile gloves	100.0%	100.0%	88.9%	96.3%	98.8%
	Handwashing materials	100.0%	100.0%	83.3%	96.3%	94.0%
	Guidelines for referral of small or sick newborn	90.9%	44.4%	22.2%	27.8%	39.3%

Figure S6. Small and sick newborn care readiness scores (%) by facility type and managing authority

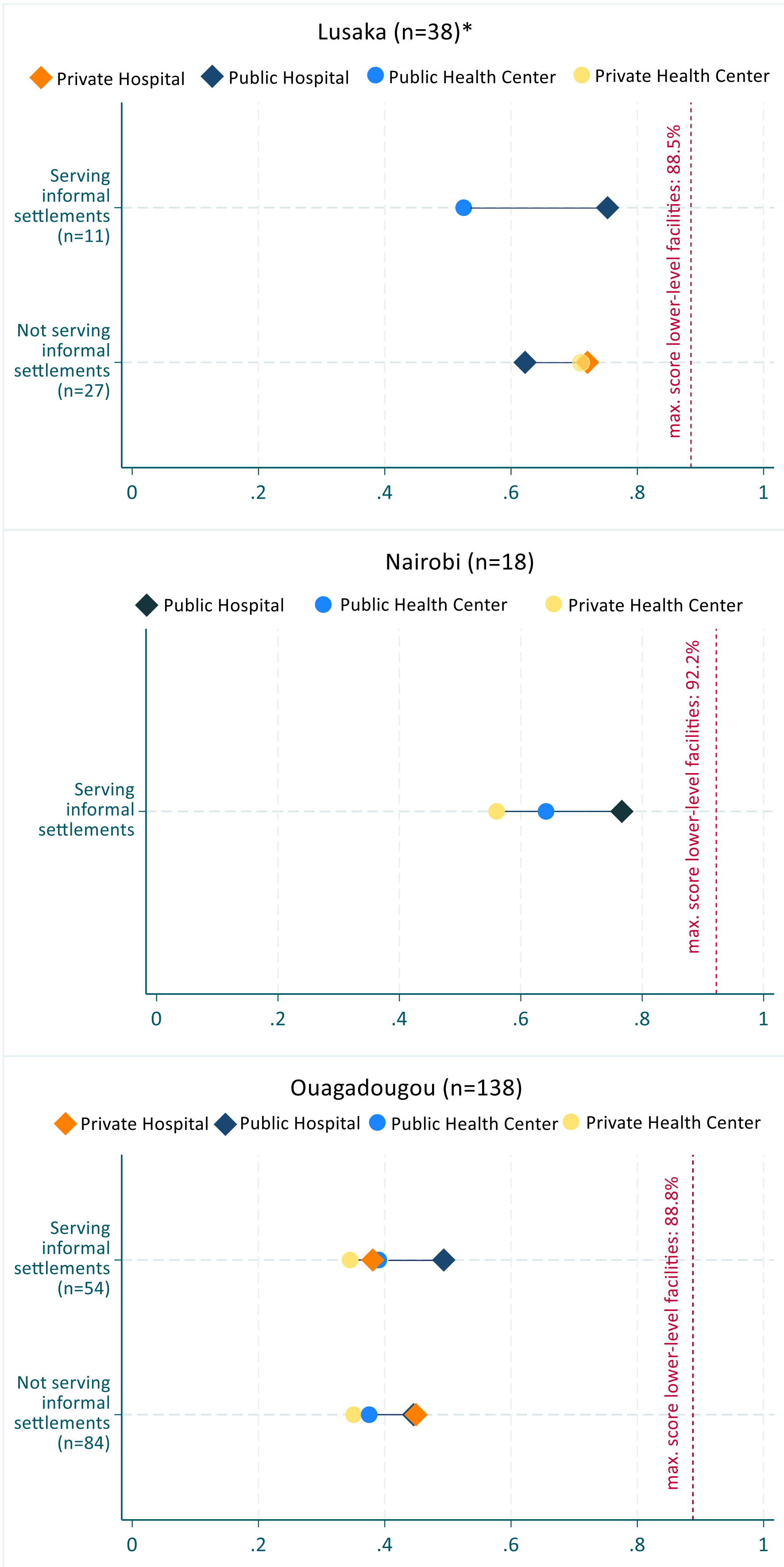

**in Lusaka there were no private facilities serving informal settlements, and there were no public health centers in areas not serving informal settlements; in Nairobi there were no private hospitals serving informal settlements.*

Note: The red dashed line represents the maximum score for lower-level facilities, based on exclusion of infant incubator, resuscitation table, x-ray machine, and morphine, and was calculated at 88.5% in Lusaka, 92.2% in Nairobi and 88.8% in Ouagadougou.

Table S9. Characteristics of women included in exact match linking analysis in Nairobi, Lusaka and Ouagadougou.

	Lusaka (n=424)			Nairobi (n=411)			Ouagadougou (n=401)		
**Women's characteristics**	%	SD	n	%	SD	n	%	SD	n
Age group			424			411			401
15-19 yrs	11.1%	31.4		6.1%	23.9		6.7%	25.1	
20-34 yrs	76.9%	42.2		82.7%	37.8		80.0%	40.0	
35-49 yrs	12.0%	32.6		11.2%	31.6		13.2%	33.9	
Education category			424			411			401
None	2.8%	16.6		6.6%	24.8		42.1%	49.4	
Primary	25.5%	43.6		24.6%	43.1		25.4%	43.6	
Secondary +	71.7%	45.1		68.9%	46.4		32.4%	46.9	
Marital status									
In union	79.0%	40.7		87.1%	33.5		98.5%	12.1	
Not in union	21.0%	40.7		12.9%	33.5		1.5%	12.1	
Employment status			424			411			401
Not employed/no income	57.3%	49.5		63.0%	48.3		48.6%	50.0	
Employed (public, private, self)	33.7%	47.3		23.1%	42.2		17.2%	37.8	
Informal/casual labor	9.0%	28.6		13.9%	34.6		34.2%	47.5	
Parity			423			411			401
1	27.7%	44.8		28.7%	45.3		19.7%	39.8	
2-3	49.9%	50.1		59.6%	49.1		52.6%	50.0	
4+	22.5%	41.8		11.7%	32.2		27.7%	44.8	
Facility type & managing authority			424			411			401
Public Health Center	45.8%	49.9		23.8%	42.7		52.6%	50.0	
Private Health Center				23.4%	42.4		17.0%	37.6	
Public Hospital	54.2%	49.9		52.8%	50.0		13.2%	33.9	
Private Hospital				0.0%			17.2%	37.8	
Number of ANC contacts			424			410			380
0	1.4%	11.8		1.0%	9.8		2.6%	16.0	
1-3	30.0%	45.9		32.7%	47.0		28.7%	45.3	

4+	68.6%	46.5		66.3%	47.3		68.7%	46.4	
Provider assisting at birth (highest level reported)			424			411			401
Physician/specialist	10.1%	30.2%		43.3%	49.6		2.5%	15.6	
Midwife, TBA, nurse	86.1%	34.7%		54.7%	49.8		55.1%	49.8	
Other/unskilled	0.9%	9.7%					0.5%	7.1	
Don't Know	2.8%	16.6%		1.9%	13.8		41.9%	49.4	
Private bed for labor/delivery			424			411			401
No	14.6%	35.4%		11.7%	32.2		10.0%	30.0	
Yes	85.4%	35.4%		88.3%	32.2		87.3%	33.4	
Don't know							2.7%	16.4	
Accompanied by partner/family during labor/delivery			424			411			401
No	98.3%	12.8%		95.9%	19.9		57.9%	49.4	
Yes	1.7%	12.8%		4.1%	19.9		39.7%	49.0	
Don't know							2.5%	15.6	
**Length of stay in facility**			424			411			401
<24h	73.3%	44.3%		16.1%	36.8		65.8%	47.5%	
≥24h	26.7%	44.3%		83.9%	36.8		34.2%	47.5%	
Maternal PNC check before discharge			419			410			398
No	5.5%	22.8%		4.4%	20.5		10.1%	30.1	
Yes	94.5%	22.8%		95.6%	20.5		89.9%	30.1	
Newborn PNC check before discharge			419			410			399
No	8.1%	27.3%		5.9%	23.5		12.8%	33.4	
Yes	91.9%	27.3%		94.1%	23.5		87.2%	33.4	

Table S10. Labor and delivery readiness scores (%) in facilities included in exact match linking analysis in Nairobi, Lusaka and Ouagadougou.

**Domain**	**LUSAKA Serving study area (n=10)**	**NAIROBI Serving study area (n=16)**	**OUAGADOUGOU Serving study area (n=54)**
Overall labor & delivery readiness *(simple additive score)*	73.7%	72.7%	59.8%
Equipment	79.1%	67.9%	64.1%
Medicines & commodities	60.8%	76.9%	50.3%
Basic amenities	80.0%	79.7%	72.2%
Human resources & guidelines	74.3%	71.4%	44.2%
Performance of BEmONC signal function in last 12 months	75.7%	77.7%	73.3%
% facilities with score >=95% (ceiling)	*10.0%*	*0.0%*	*0.0%*
% facilities with score <=5% (floor)	*0.0%*	*0.0%*	*0.0%*

Table S11. Associations between facilities' labor & delivery care readiness scores (%) and women's PCMC domain scores in study areas by city.

	**Crude model**			**Adjusted Model**		
	Coefficient [95%CI]	P value	n	Coefficient [95%CI]	P value	n
**DIGNITY AND RESPECT (18 points)**						
Lusaka+	-0.03 [-0.08, 0.03]	0.299	424	-0.02 [-0.07, 0.03]	0.370	410
Nairobi++	0.01 [-0.03, 0.05]	0.684	411	0.01 [-0.02, 0.04]	0.376	406
Ouagadougou+++	0.02 [-0.02, 0.06]	0.265	401	0.03 [-0.01, 0.07]	0.152	373
**COMMUNICATION & AUTONOMY (27 points)**						
Lusaka+	-0.003 [-0.13, 0.12]	0.965	424	-0.07 [-0.29, 0.15]	0.536	410
Nairobi++	-0.06 [-0.14, 0.15]	0.115	411	**-0.06 [-0.12, -0.01]**	**0.027**	407
Ouagadougou+++	-0.01 [-0.07, 0.05]	0.732	401	0.01 [-0.05, 0.07]	0.723	371
**SUPPORTIVE CARE (45 points)**						
Lusaka+	-0.11 [-0.23, 0.01]	0.081	424	**-0.22 [-0.37, -0.07]**	**0.004**	408
Nairobi++	-0.01 [-0.13, 0.11]	0.869	411	**0.08 [0.01, 0.15]**	**0.030**	406
Ouagadougou+++	0.04 [-0.05, 0.14]	0.381	401	0.04 [-0.05, 0.13]	0.407	394

Notes:

All adjusted models included variables significant at $p<0.2$ in bivariate association with PCMC domain scores:

+ Lusaka:

Dignity and respect model adjusted by maternal PNC check received before discharge, PNC counseling on danger signs received before discharge, PNC, blood pressure checked before discharge, newborn PNC check received before discharge, appointment received for next PNC check before discharge; Communication and autonomy model adjusted by: facility type and managing authority, women's education, women's employment status, women's age, history of pregnancy complications, type of provider assisting during delivery, maternal PNC check received before discharge, PNC counseling on danger signs received before discharge, PNC counseling on family planning received before discharge, blood pressure checked before discharge, newborn PNC check received before discharge; Supportive care model adjusted by: facility type and managing authority, parity, type of provider assisting during delivery, maternal PNC check received before discharge, PNC counseling on danger signs received before discharge, PNC counseling on family planning received before discharge, blood pressure checked before discharge, newborn PNC check received before discharge, appointment received for next PNC check before discharge.

++ Nairobi:

Dignity and respect model adjusted by delivery facility type and managing authority, women's employment, women's age, type of provider assisting during delivery, maternal PNC check received before discharge, PNC counseling on danger signs received before discharge, blood pressure checked before discharge, newborn PNC check received before discharge, appointment received for next PNC check before discharge; Communication and autonomy model adjusted by: facility type and managing authority, women's employment status, women's marital status, place of ANC, type of provider assisting during delivery, length of facility stay, PNC counseling on danger signs received before discharge, PNC counseling on family planning received before discharge, blood pressure checked before discharge, newborn PNC check received before discharge; Supportive care model adjusted by: facility type and managing authority, women's education, women's age, marital status, type of provider assisting during delivery, maternal PNC check received before discharge, PNC counseling on danger signs received before discharge, PNC counseling on family planning received before discharge, blood pressure checked before discharge, newborn PNC check received before discharge, appointment received for next PNC check before discharge.

+++ Ouagadougou:

Dignity and respect model adjusted by delivery facility type and managing authority, women's marital status, parity, ANC4+, type of provider assisting during delivery, maternal PNC check received before discharge, PNC counseling on danger signs received before discharge, PNC counseling on family planning received before discharge, blood pressure checked before discharge, newborn PNC check received before discharge, appointment received for next PNC check before discharge; Communication and autonomy model adjusted by: facility type and managing authority, women's employment status, history of pregnancy complications, ANC4+, type of provider assisting during delivery, maternal PNC check received before discharge, PNC counseling on danger signs received before discharge, PNC counseling on family planning received before discharge, blood pressure checked before discharge, newborn PNC check received before discharge, appointment received for next PNC check before discharge; Supportive care model adjusted by: facility type and managing authority, history of miscarriage/stillbirth, type of provider assisting during delivery, maternal PNC check received before discharge, length of stay in facility, PNC counseling on danger signs received before discharge, PNC counseling on family planning received before discharge, blood pressure checked before discharge, newborn PNC check received before discharge.
